# Selection-Driven Extinction Dynamics for Group II Introns in *Enterobacteriales*

**DOI:** 10.1371/journal.pone.0052268

**Published:** 2012-12-14

**Authors:** Sébastien Leclercq, Richard Cordaux

**Affiliations:** Université de Poitiers, CNRS UMR 7267 Ecologie et Biologie des Interactions, Poitiers, France; Louisiana State University, United States of America

## Abstract

Transposable elements (TEs) are one of the major driving forces of genome evolution, raising the question of the long-term dynamics underlying their evolutionary success. Some TEs were proposed to evolve under a pattern of periodic extinctions-recolonizations, in which elements recurrently invade and quickly proliferate within their host genomes, then start to disappear until total extinction. Depending on the model, TE extinction is assumed to be driven by purifying selection against colonized host genomes (Sel-DE model) or by saturation of host genomes (Sat-DE model). Bacterial group II introns are suspected to follow an extinction-recolonization model of evolution, but whether they follow Sel-DE or Sat-DE dynamics is not known. Our analysis of almost 200 group II intron copies from 90 sequenced *Enterobacteriales* genomes confirms their extinction-recolonization dynamics: patchy element distributions among genera and even among strains within genera, acquisition of new group II introns through plasmids or other mobile genetic elements, and evidence for recent proliferations in some genomes. Distributions of recent and past proliferations and of their respective homing sites further provide strong support for the Sel-DE model, suggesting that group II introns are deleterious to their hosts. Overall, our observations emphasize the critical impact of host properties on TE dynamics.

## Introduction

Transposable elements (TEs), which are mobilizing pieces of DNA, are widely distributed in eukaryote and prokaryote genomes, and they may represent substantial fractions of the genomes, as in *Homo sapiens* [1] or *Zea Mays* [2]. TEs are major drivers of genome evolution, sometimes as actors of genetic innovation, or by creating genomic instability or genetic disorders [3]–[5]. Given their relative deleteriousness, their tremendous evolutionary success results from a complex interplay between transposition rate, fitness cost, and host effective population size [6]–[10]. In multicellular eukaryotes, the rather low effective population size (compared to unicellular organisms) induces a reduction of selection efficiency and enhanced genetic drift, so that slightly deleterious TE insertions may be maintained [7], [11]. Therefore, copies accumulate in nearly neutral genomic regions and undergo gradual degradation, which results in a large amount of old, non functional copies and few but still active elements [1], [12], [13].

By contrast, prokaryotes usually have much larger effective population sizes, and studies on insertion sequence (IS) TEs suggested that very few TE remnants were present within genomes while most active elements resulted from recent acquisitions and proliferations [14]–[16]. To explain the evolutionary success of IS elements in prokaryotes, a periodic extinction-recolonization model has been proposed, in which bacterial genomes undergo recurrent TE acquisitions and proliferations, followed by rapid elimination of the resulting TE copies [17]. This scenario has recently received direct empirical evidence, based on the analysis of the unusual IS fossil record buried in the genomes of the bacterial endosymbiont *Wolbachia* [18]. The model is driven by two antagonist properties of IS elements: their transposition rate and their deleteriousness to the host. After acquisition by an IS-free host through horizontal transfer, a novel active IS element proliferates in the host genome due to its high transposition rate. IS copies may then invade the population through multiplication of the colonized cell or IS transmission to neighboring cells through intra-population horizontal transfers. However, genomes harboring IS elements are rapidly removed from the bacterial population because of the deleterious effect of IS insertions, and the bacterial population finally goes back to its initial IS-free state. The second and third steps heavily depend on IS and host genetic properties, and host population sizes [5], [18]–[20], such that most newly acquired IS are probably removed from the population even before having a chance to proliferate. In that way, a high rate of acquisition is critical to ensure IS persistence in prokaryotes [21]. In prokaryotes, TE acquisition is thought to be mainly achieved through larger mobile genetic elements (MGEs), especially plasmids which carry numerous IS elements [22].

Such dynamics (accumulation in neutral genomic regions or periodic extinctions-recolonizations) are largely driven by the deleteriousness of TEs, and radically different dynamics are expected for TEs which are not constrained by host selective pressures. For instance, group I introns are genetic elements mainly found in housekeeping genes of eukaryote organelles and prokaryotes, which are able to self-splice from the transcript they are located in, making them silent at the translational level [23]. Most of them harbor a homing endonuclease gene that allows them to duplicate from an occupied locus to a highly-related intron-free locus (mainly another allele of the same gene) through a process called homing [23]. According to their very high site specificity and self-splicing ability, group I intron insertions are likely to be largely neutral, and a model of recurrent invasions and extinctions was proposed for group I introns in organelles and chromosomes of unicellular eukaryotes [24], [25]. In this model, a newly acquired element can spread in the population through homing during mating (when infected and free alleles are together in a unique cell) until fixation (*i.e.* no free allele available in the population). When the population is saturated, the element cannot home anymore and is slowly degraded through point mutations, to finally lead to static, splicing-only copies. These remnants are sometimes precisely removed, reconstructing a free homing site, which becomes available for future group I invasion.

Group II introns are TEs related to non-long terminal repeat retrotransposons, found in organellar and prokaryote genomes [26], and the putative ancestors of spliceosomal introns in eukaryotes [27], [28]. Similar to group I introns, group II introns are catalytic RNAs which are able to self-splice from the transcript they are inserted in. The molecular splicing mechanism requires base-pairing interactions between three short motifs located on the intron RNA (EBS1 to 3, for exon-binding sites) and their complementary motifs on the transcript RNA (IBS1 to 3, for intron-binding sites) spanning positions from −12 to +1 relative to the intron insertion site [29], [30]. Group II introns move via a target-primed reverse transcription mechanism called retrohoming, which is processed by the intron-encoded protein (IEP) [30], [31]. The IEP possesses several catalytic domains necessary for intron mobility, such as reverse-transcriptase (RT) and maturase (X) domains, and sometimes an endonuclease (En) domain. When produced, the IEP binds to the intron RNA and helps in its efficient splicing using the maturase activity, resulting in a free ribonucleoprotein (RNP) particle composed of the intron ribozyme and the IEP. The RNP complex then recognizes a new insertion site through interactions between the IEP and a small number of specific nucleotides in the distal 5'-exon region of the target site, in addition to EBS-IBS binding [32]–[34]. The intron RNA is then reverse-spliced at the integration position using these IBS-EBS interactions, and finally reverse-transcribed by the IEP [30]. Such a specific targeting ensures integration of group II introns primarily in intron-free alleles of the same gene, although some cases of non-specific retrotransposition have been reported, involving only (sometimes imperfect) IBS motifs [35]–[37]. Some group II introns, called bacterial class C introns, also shift from this general pattern, as they harbor only two EBS (EBS1 and EBS3) and they specifically insert downstream of Rho-independent transcription terminators [30], [38].

Group II introns are widespread in the bacterial kingdom and show a surprising diversity. They are separated into three ribozyme groups (IIA, IIB, and IIC) and nine ORF classes (A to F, CL1, CL2, ML) which may have diverged for several hundred million years [39]. Moreover, studies conducted on natural populations/species from different bacterial groups revealed an extreme variability in group II intron abundance and diversity between bacterial strains [40]–[44], suggesting recurrent extinction-recolonization dynamics for these elements. According to group II intron properties (site specificity and self-splicing), we would expect a model of dynamics not constrained by host selection, *i.e.* rapid homing site saturation in the population, followed by slow sequence degradation and removal, similar to that of eukaryote group I introns. However, several observations suggest that group II introns may not be selectively neutral in bacteria. First, they are virtually never found in housekeeping genes [26], [45]. Rather, they are preferentially found inserted into regions non essential for the bacterial host, such as plasmids and other mobile genetic elements [42], [45]–[48]. In addition, some empirical evidence indicates very low efficiency to render viable exons after splicing [49], [50]. Finally, group II introns are sources of genomic instability in some bacteria [43]. These observations thus suggest that bacterial group II intron dynamics may be governed by host selective pressures, similar to the extinction-recolonization model proposed for IS elements.

**Figure 1:**
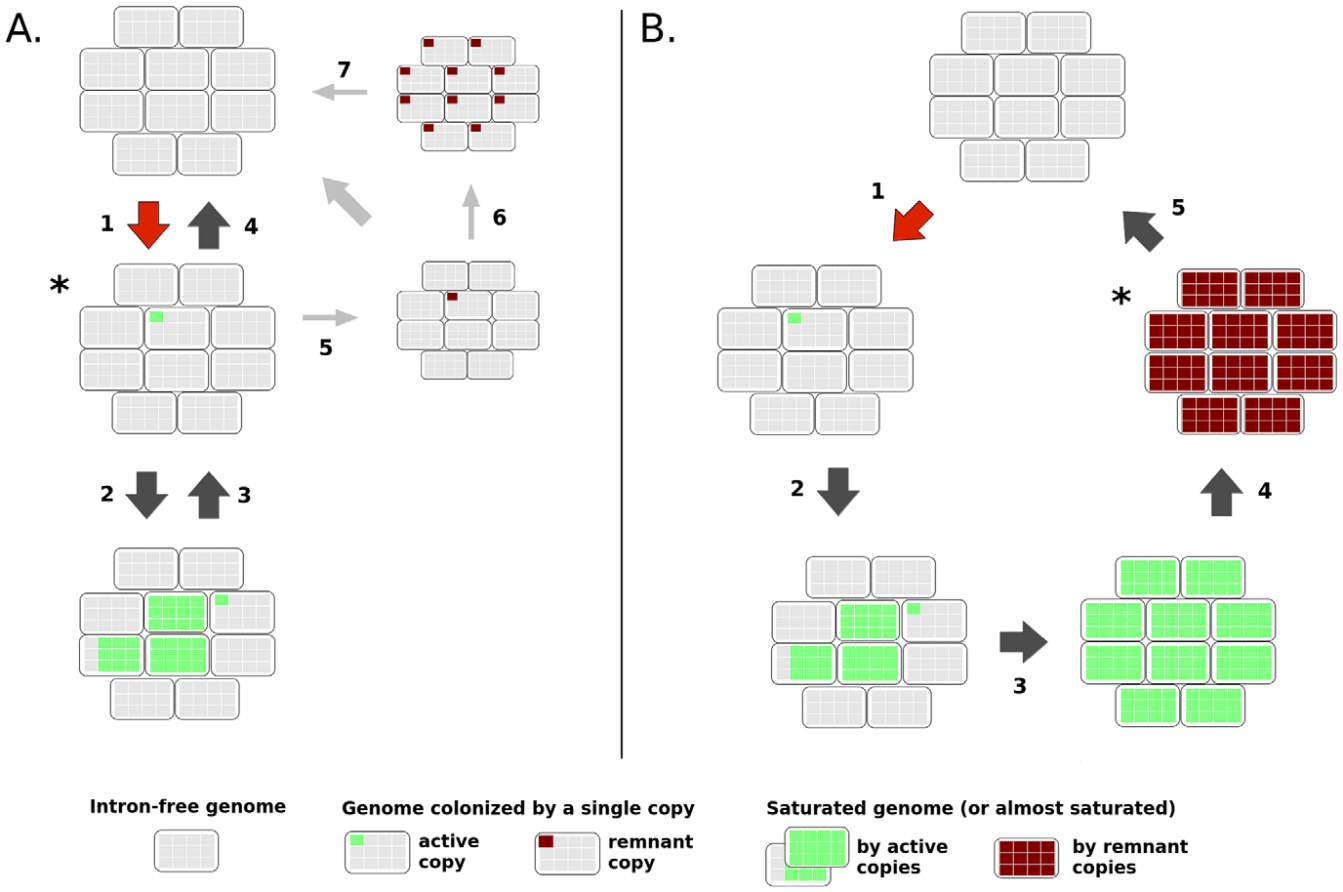
Two proposed extinction-recolonization dynamics of group II introns in a bacterial population. (**A**) Selection-driven extinction (Sel-DE) model: acquisition of a functional copy via a MGE-mediated horizontal transfer (step 1, red arrow), quick element proliferation in the host genome and in the population (step 2), and removal of bacterial individuals with proliferated copies through purifying selection (step 3). Individuals with few copies ultimately disappear from the population through rampant purifying selection or eventually genetic drift, resulting in complete loss of the group II intron from the bacterial population (step 4). Alternatively, not proliferated copies may be inactivated by mutation (step 5), evolve as neutral genomic regions and eventually become fixed in the population (step 6). Residual inactivated copies then undergo slow degradation until complete loss from the bacterial genome (step 7). (**B**) Saturation-driven extinction (Sat-DE) model: the first two steps are identical to those of the Sel-DE model, but proliferation results in the saturation of all available homing sites in the population (step 3). Proliferated copies then start to degenerate (step 4) and are slowly removed from the population through random mutations and deletions (step 5). Bacterial populations are represented by sets of cells (rounded rectangles) harboring available homing sites (grey boxes) or homing sites occupied by active (green boxes) or inactive intron copies (dark red boxes). The starred state in each model is those predicted to be the most frequently observed when a group II intron copy is present. Smaller cartoon sizes (in **A**) reflect infrequent alternative.

Applied to group II introns, these models can be summarized as proposed in Figure 1: Colonization always starts with an acquisition of a novel active element by one cell in an element-free bacterial population, which then spreads in the host genome and in the population. A selection-driven extinction (Sel-DE) model then predicts that highly colonized genomes are removed from the population through purifying selection (Figure 1A), while a saturation-driven extinction (Sat-DE) model predicts a saturation of all available homing sites in the host population without elimination of highly colonized genomes (Figure 1B). Resulting copies are finally inactivated and degraded until their complete loss. The two models are expected to lead to critical differences in terms of observed group II intron distributions within genomes. Under the Sel-DE model, most genomes should be free of group II introns; and when present, elements should be mostly active and observed at very low copy number. Genomes saturated by active or degraded elements can be rarely observed. Under the Sat-DE model, we expect mainly degraded elements located in all potential homing sites, and probably shared between closely related strains. Few genomes at transient state with a variable number of active copies may also be observed. Finally a number of genomes should also be free of group II introns, depending on the probability of element acquisition.

To identify the evolutionary forces driving group II intron dynamics in bacteria, we analyzed their abundance and distribution in 90 complete genomes from *Enterobacteriales* (*i.e. Escherichia coli* and relatives) and compared our observations to expectations under the Sel-DE and Sat-DE models. First, we provide clear examples of plasmid-mediated acquisition and rapid, strain-specific proliferations, consistent with the extinction-recolonization behavior already suspected for group II introns. The distribution of recent and more ancient proliferations also support the Sel-DE model, in which group II intron extinctions are driven by removal of overloaded genomes from the population though purifying selection. Finally, our data highlight the impact of bacterial lifestyle on group II intron abundance and evolution.

## Materials and Methods

### Genome Sequences

Complete sequences of the 90 *Enterobacteriales* genomes available in GenBank as of September 14, 2009 were downloaded from the GenBank FTP site (ftp://ftp.ncbi.nih.gov/genomes/Bacteria/all.fna.tar.gz); they are listed in Table S1. Genomes were grouped into 18 clusters according to their genus name, except *Shigella* and *Escherichia* strains which belong to the same complex [51]–[53], and *Erwinia* and *Pectobacterium* strains which are also included in the same complex [54], [55].

### Group II Intron Detection

We downloaded the 397 reference sequences of putatively functional introns available in Zimmerly's group II intron database (update July 29, 2011), hereafter referred to as Zbase [56], and constructed nucleotide and protein libraries. The nucleotide library contained whole group II intron sequences while the protein library contained the amino acid residues encoded by their open reading frame (ORF). We complemented these datasets with sequences of full-length but putatively non-functional introns that were available in a previous Zbase version (update March 11, 2008). BLAST searches were then performed for nucleotide and protein intron libraries against *Enterobacteriales* genomes (e-value <0.01), and results were filtered for overlapping detections to keep hits with the highest number of identical sites (nucleotides or amino acid residues). Detections shorter than 100 bp in length or less than 75% similarity to the reference sequence (for BLASTN only) were discarded to avoid false positives. Group II intron copies were categorized as full-length when their extremities could be unambiguously defined, and fragmented otherwise. For copies that did not cover the whole sequence of their intron reference, boundaries were searched by comparing flanking sequences of multiple instances of the same intron (when available), or by looking for an intron-free locus homologous to the insertion site in GenBank. A second round of BLASTN searches using all previously detected copies (full-length and fragmented copies) was performed to search for more divergent copies. Intron names were attributed according to the Zbase nomenclature for copies showing more than 10% nucleotide divergence compared to their reference intron sequence, and with the Zbase reference intron name otherwise.

### Determination of Insertion Patterns and Orthology Relationships

Insertion patterns and orthology relationships were evaluated using the following comparison procedure. The immediate 3 kbp flanking sequences of each intron copy were searched against other genomes of the same genus/complex using BLASTN. When the 5′ and 3′ flanks aligned to directly adjacent regions in another genome, the copy was considered to result from retrohoming in the query genome. When 5′ and/or 3′ flanks of an intron copy aligned with the 5′ and/or 3′ flanks of an intron copy in another genome, copies were considered as orthologous, with a few exceptions (see below). For analyses of abundance of distinct elements, orthologous copies were counted as only one element. Flanking sequences of many group II intron copies were not readily identifiable using this procedure, mainly because they were parts of larger MGEs, such as integrated plasmids, genomic islands (GIs), or prophages. For these intron copies, the surrounding genomic region (up to 1 Mbp) was compared visually to closely related genomes, using the MaGe synteny browser [57]. In most cases, this analysis revealed specific insertions of large genetic sequences (>10 Kbp), which were considered as integrated MGEs. During this analysis, we found that the SelC intron-carrying GI inserted independently at two different genomic loci in two *E. coli* lineages (CFT073/ED1a and S88/APEC_01). Thus, the group II intron copies carried by these two GIs were considered as distinct, while they were initially considered as orthologous with the 3 Kbp flanking sequence comparison (see above). Two other cases of shared but not vertically inherited GIs were detected: between the 55989 *E. coli* strain and the *Shigella flexneri* lineage, and between the two *E. coli* strains ED1a and 536. Group II intron copies inserted in these GIs were consistently counted as distinct copies. Insertion patterns, positions and orthology relationships are shown in Table S2 and Table S3.

### Detection of Putative Available Homing Sites

Four major schemes were conducted to detect potential group II intron homing sites. For introns which retrohome into specific IS elements (*E.c*.I1, *E.c*.I3, *E.c*.I4, *E.c*.I9, and c-*Ha.de*.I1 subgroup 2), the 90 bp regions surrounding homing sites were searched within genome sequences using a BLASTN procedure. Hits with a e-value ≤0.0001, a similarity ≥90% with the reference 90 pb, and that overlapped with the precise integration site were counted as putative homing sites. For other already referenced introns (*Kl.pn*.I5, *Di.ze*.I1, and *So.gl*.I1), putative EBS motifs were retrieved from the secondary structure available on the current (http://webapps2.ucalgary.ca/~groupii/index.html) or previous (http://www.fw.ucalgary.ca/group2introns/index.htm) versions of Zbase. Consensus IBS sequences were constructed by aligning the predicted EBS motifs to the 5′ and 3′ regions surrounding detected full-length copies. For *Kl.pn*.I5, sequence surrounding the Zbase copy was also added to the analysis, and all identical bases spanning from positions −25 to +10 relative to the insertion site were added to the consensus homing site sequence. Consensus sequences were then searched within genomes using a Perl script according to standard base-pairing rules with perfect match. For introns belonging to bacterial ORF class C (*Di.ze*.I1 and *So.gl*.I1), host genomes were searched for Rho-independent transcription terminators with the ARNold algorithm [58]. Putative IBS1/3 located 3' to a transcription terminator stem-loop were considered as potential homing sites. Finally, for c-*Ha.de*.I1 subgroup 1 for which EBS motifs are unknown, conserved bases flanking the 26 full-length copies were estimated using Weblogo 3.3 [59]. The estimated consensus sequence was then searched within the *H. defensa* genome using the same Perl script as above.

### Computation of Intra-genus Mean Nucleotide Divergence

We estimated intra-genus mean nucleotide divergence for the *Escherichia/Shigella* complex and the *Salmonella* genus as a measure of genus diversity given by the sequenced strains. For each genus/complex, we searched all genomes for the housekeeping genes used for MLST analyses, according to the MLST database (http://mlst.ucc.ie/): *adk, fumC, gyrB, icd, mdh, purA* and *recA* for *Escherichia/Shigella* genomes, and *aroC, dnaN, hemD, hisD, purE, sucA* and *thrA* for *Salmonella* genomes. Gene sequences were concatenated and aligned with the L-INS-i method of MAFFT [60], and mean nucleotide divergence was computed for each genus/complex with MEGA 5 [61] using third codon positions only. Genomes of *E. fergusonii* and *S. enterica* serovar *arizonae* can be considered as outgroups compared to other *Escherichia/Shigella* and *Salmonella* genomes respectively [53], [62], and were excluded from the analysis.

## Results

### General Abundance and Distribution of Group II Introns in Enterobacteriales

**Figure 2:**
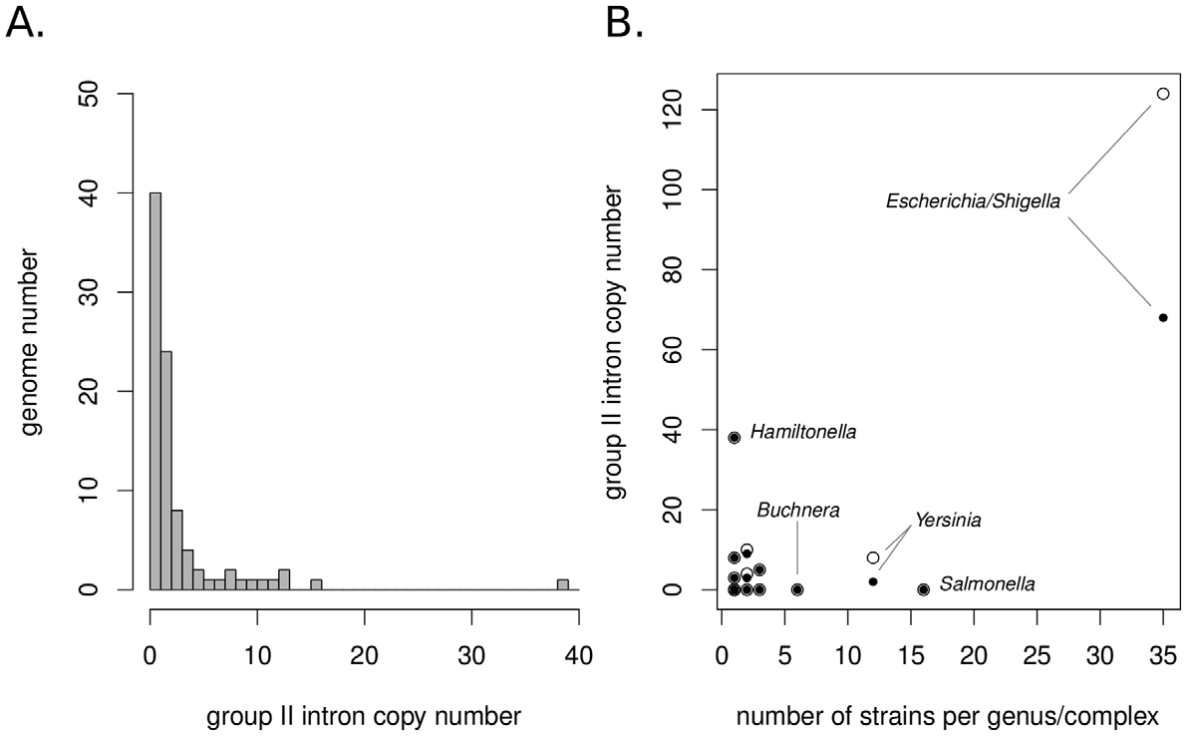
Distribution of (A) 90 *Enterobacteriales* genomes (corresponding to 18 genera/complexes) according to their group II intron abundance, and (B) group II intron abundance according to sequenced strain number within genera/complexes. White dots: total number of detections considered; black dots: only distinct copies considered (see text). Names of genera/complexes with >20 intron copies and/or >5 sequenced strains are indicated.

We searched 90 sequenced genomes of *Enterobacteriales* for group II intron elements and found a total of 198 copies longer than 100 bp (see Methods), 87 (44%) of which are full-length elements. We found that 132 (67%) copies are inserted in chromosomes and 66 (33%) in plasmids. Although it represents a density of 2.2 group II intron copies per genome on average, only 50 (56%) of the 90 analyzed genomes carry at least one group II intron (Figure 2A). Moreover, group II introns are not uniformly distributed among the different bacterial genera, and their abundance does not seem to be related to the number of sequenced genomes within genera (Figure 2B). Indeed, there was no significant relationship between the number of sequenced strains per genus/complex and the abundance of group II introns (Chi square test, *P* = 0.41). We searched for orthologous copies within genera/complexes to prevent counting several times copies that originated from a single ancestral insertion event, which would artificially inflate the overall abundance of group II introns in genera with more than one sequenced strain (see Materials and Methods). A total of 90 copies were found to be orthologous between at least two genomes within genera, reducing the total number of detections to 135 distinct intron copies (*i.e.* when orthologues are counted as a single copy; Table 1). Again, the relationship between the number of sequenced strains per genus/complex and the abundance of distinct copies was not significant (Chi square test, *P* = 0.23).

**Table 1:** Distribution of the 198 detected group II intron copies among the 18 *Enterobacteriales* genera/complexes.

Bacterial genus	Number ofstrains	Number ofcopies	Distinctcopies	Full-lengthdistinct copies	Distinctfragments
*Escherichia/Shigella*	35	122	**67**	40*	27
*Hamiltonella*	1	38	**38**	30	8
*Photorabdus*	2	10	**9**	2	7
*Sodalis*	1	8	**8**	3	5
*Klebsiella*	3	5	**5**	2	3
*Dickeya*	2	4	**3**	3	0
*Serratia*	1	3	**3**	0	3
*Yersinia*	12	8	**2**	0	2
*Buchnera*	6	0	**0**	0	0
*Blochmania*	2	0	**0**	0	0
*Citrobacter*	1	0	**0**	0	0
*Cronobacter*	1	0	**0**	0	0
*Edwarsiella*	1	0	**0**	0	0
*Enterobacter*	1	0	**0**	0	0
*Pectobacterium/Erwinia*	3	0	**0**	0	0
*Proteus*	1	0	**0**	0	0
*Salmonella*	16	0	**0**	0	0
*Wigglesworthia*	1	0	**0**	0	0
**TOTAL**	**90**	**198**	**135**	**80**	**55**

*: Two orthologous copies in the *Escherichia/Shigella* complex are full-length in some strains and fragmented in others; they are counted only as distinct full-length copies.

Among the four most sequenced genera (*i.e.* >5 sequenced strains), only the *Escherichia/Shigella* complex shows group II intron abundance exceeding two distinct copies (Figure 2B). The absence of group II intron in the six *Buchnera aphidicola* strains is expected, as these bacteria are ancient mutualistic endosymbionts with highly reduced genomes known to be completely devoid of TEs [63]. Concerning the *Yersinia* genus, 11 of the 12 sequenced strains belong to the very closely related and almost monomorphic *Y. pestis* and *Y. pseudotuberculosis* species [64], [65]. These data therefore probably display only a small subset of the whole *Yersinia* diversity, which may account for the apparent paucity of group II intron in this genus. Sequenced *Salmonella* strains also represent a small subset of the whole genus diversity, as all strains (except *S. enterica arizonae*) belong to the *S. enterica* group I [62]. However, the sequenced *Salmonella* strains show a genetic diversity nearly equivalent to that of *Escherichia/Shigella* strains (mean nucleotide divergence on MLST genes of 0.027 and 0.038, respectively), and they have experienced several DNA acquisitions through plasmids and prophages since their divergence [62]. The complete lack of group II intron detection in the 16 *Salmonella* genomes may therefore be attributed to the erratic nature of extinction-recolonization processes [21].

### Group II Intron Acquisition through MGE-mediated Horizontal Transfers

In both Sel-DE and Sat-DE models, horizontal transfers of group II intron copies between bacterial cells are essential to ensure periodic recolonizations. Such transfers are believed to occur via large MGEs such as plasmids or GIs [42], [46]–[48]. Genomic locations of group II introns in the *Escherichia/Shigella* complex support a high horizontal transfer potential, as 54 (81%) of the 67 distinct *Escherichia/Shigella* group II intron copies are located in free or integrated MGEs (Table S2). Although some intron copies may have inserted in MGEs after MGE acquisition by host bacterial cells, instead of being shuttled by them, we identified at least three highly probable cases of group II intron horizontal transfers via MGEs in *Escherichia/Shigella* strains. The first transfer concerns an *E.c*.I3 copy in *E. coli* strains ED1a and 536 (unique *E.c*.I3 copy in this strain), which is located at an orthologous position in the same GI (PAI1), but this GI is located at unrelated positions in the two bacterial strains. Similarly, *E.c*.I6 is present only once in *E. coli* strains CFT073, ED1a, S88, and APEC_O1, and is inserted at an orthologous site in a GI inserted at different loci in the various strains. Finally, the SHI-1 GI carries a *E.c*.I4 intron copy at the same position in the two *Shigella flexneri* 2a strains (the only full-length copy in these strains) and in *E. coli* 55989, but the GI is again inserted at different loci.

These examples show group II intron acquisitions following chromosomal integration of the host MGE. In addition, other *Enterobacteriales* genomes provide us with strong support for chromosomal acquisition through another mechanism, namely MGE-to-chromosome retrohoming. For example, there are two full-length *Kl.pn*.I5 intron copies in the *Klebsiella pneumoniae* MGH genome: one located in a plasmid and the other one showing evidence of retrohoming in the chromosome. Both intron copies are identical at the nucleotide level, suggesting that the chromosomal copy was acquired from the plasmid through retrohoming (although a transfer from the chromosome to the plasmid cannot be formally ruled out). *Dickeya* genomes provide another example of MGE-to-chromosome intron acquisition. One of these genomes carries two *Di.ze*.I1 copies, which are identical and were acquired through retrohoming (as both homing sites are free in the other *Dickeya* genome). As no other *Di.ze*.I1 copy was detected in this genome, observed copies were most probably acquired from a non-integrated MGE which has since disappeared.

### Homing Site Occupation of Newly Acquired, Potentially Active Group II Introns

Following acquisition, both models predict that incoming elements start to proliferate, sometimes quite rapidly for group II introns [43], [66]. Two main factors limit group II intron proliferation: the number of available homing sites and, in the Sel-DE model, the power of selection. Thus, genomes with saturated or almost saturated homing sites should be commonly observed under the Sat-DE hypothesis, while they should be very rarely observed under the Sel-DE hypothesis (except when the number of homing sites is low).

Our dataset contains 87 full-length copies from 14 group II introns distributed among 22 genomes (Table 2). For five of them (*E.c*.I5, *Di.da*.I1, *P.l*.I1, *P.l*.I2, and c-*Ha.de*.I2), only one distinct copy is detected and their homing site have never been described. We were thus unable to infer the number of potential homing sites for these introns. Secondly, when checking intron sequences with ORFinder (http://www.ncbi.nlm.nih.gov/projects/gorf/) for the remaining elements, we found that *E.c*.I4 copies carried by *E. coli* SMS_3_5 and *S. flexneri* 2a 2457T, as well as the four *E.c*.I6 copies, exhibit a disrupted ORF. As all of them are present in free or integrated MGE (Table S2), they may have been acquired by the host genome already inactivated and unable to proliferate. Evaluating the proportion of occupation for these introns may thus not be relevant and was not conducted.

**Table 2:** Full length group II intron copies, number of potential homing sites available in their host genomes, and proportion of homing site occupation.

Group II intron	Host genome	Copy number	Available homing sites	Proportion of occupation (%)
*E.c*.I2	*E. coli* UMN026	1	4	20
*E.c*.I3	*E. coli* ED1a	2	0	100
	*E. coli* 536	1	0	100
*E.c*.I4	*E. coli* ED1a	2	1	67
	*E. coli* 55989	6	1	86
	*E. coli* E24377A	7	0	100
	*E. coli* IAI39	15	27	36
	*E. coli* SMS_3_5	1*	/	/
	*E. coli* UTI89	1	2	33
	*E. coli* UMN026	1	6	14
	*S. flexneri* Sf301	1	42	2
	*S. flexneri* 2457T	1*	/	/
*E.c*.I5	*E. coli* EDL933	1	/	/
	*E. coli* Sakai	1	/	/
*E.c*.I6	*E. coli* ED1a	1*	/	/
	*E. coli* CFT073	1*	/	/
	*E. coli* APEC_01	1*	/	/
	*E. coli* S88	1*	/	/
*E.c*.I9	*E. coli* ATCC_8739	1	6	14
*Kl.pn*.I5	*K. pneumoniae* MGH	2	0	100
*Di.da*.I1	*D. zeae* Ech1591	1	/	/
	*D. dadanti* Ech703	1	/	/
*Di.ze*.I1	*D. zeae* Ech1591	2	17	11
*P.l*.I1	*P. luminescens TT01*	1	/	/
*P.l*.I2	*P. luminescens* TT01	1	/	/
*So.gl*.I1	*S. glossinidius* 'morsitans'	3	73	4
*c-Ha.de*.I1 (subgroup 1)	*H. defensa* 5AT	26	17	60
*c-Ha.de*.I1 (subgroup 2)	*H. defensa* 5AT	3	1	75
*c-Ha.de*.I2	*H. defensa* 5AT	1	/	/

*: inactivated copies located within free or integrated large MGE.

Among the remaining, potentially active group II intron copies, *E.c*.I2, *E.c*.I9, *E.c*.I3, and *E.c*.I4 copies detected in *Escherichia/Shigella* genomes are known to specifically target Rsh IS ( = ISEc1−5), Rsh IS at another position, IS679, and various IS3 elements, respectively [40], [67]. Both *E.c*.I2 and *E.c*.I9 are found only once in their respective genomes, in which they occupy only 20% and 14% of the potential homing sites, respectively (Table 2). *E.c*.I3 is also detected at low copy number per genome (2 and 1 copies), but no free IS679 was detected in colonized genomes (Table 2). On the contrary, *E.c*.I4 shows a large range of abundance, from 1 copy in three genomes up to 15 copies in the genome of IAI39 (Table 2). All copies show <1% intra-strain nucleotide divergence and are strain-specific (except one shared by E24377A and 55989 strains), indicating that the *E.c*.I4 intron experienced very recent and independent proliferations in these *E. coli* strains. As expected, all copies are inserted at specific positions in IS3-like transposases, namely from IS629, IS911, ISEc16, and ISEc31 elements. Searches of these four potential host genes in *E. coli/Shigella* genomes revealed that genomes of 55989 and E24377A strains are saturated or almost saturated, and 67% of the potential homing sites are occupied in ED1a (Table 2). By contrast, IAI39, UTI89, and UMN026 have less than 40% occupied homing sites, and only one copy is present in *S. flexneri* Sf301 despite 42 potentially available homing sites (Table 2).

**Figure 3:**
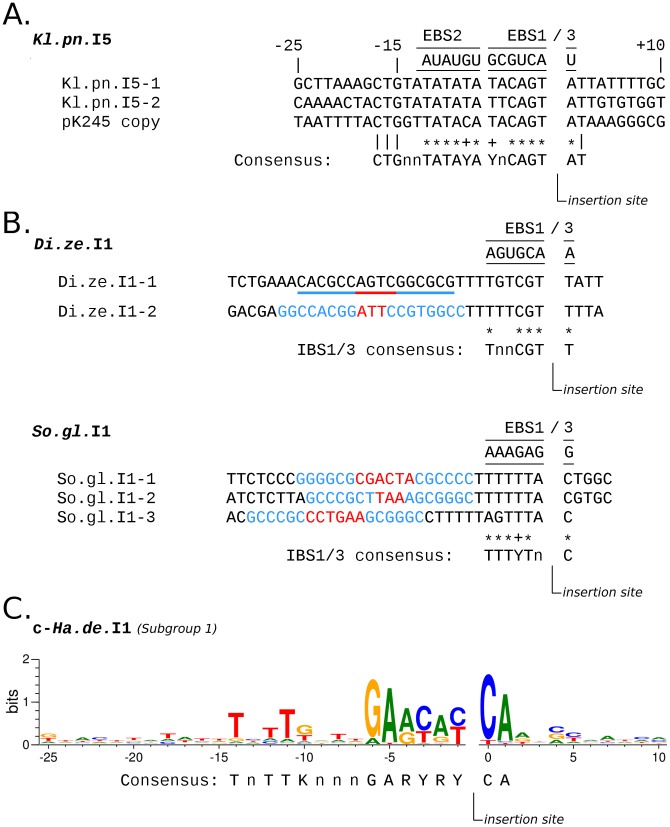
Inferred homing sites for *Kl.pn*.I5, *Di.ze*.I1, *So.gl*.I1, and c-*Ha.de*.I1 introns. (**A**) Sequence spanning from positions −25 to +10 relative to full-length *Kl.pn*.I5 copy insertion sites are displayed and compared to predicted EBS. Identical bases and those which fit the Watson-Crick (stars) or the Wobble (plus) base-pairing with EBS motifs were used to construct the consensus homing site. (**B**) Regions surrounding *Di.ze*.I1 and *So.gl*.I1 full-length copies are displayed and compared to predicted EBS1 and EBS3. Most represented nucleotides at a given position and which fit the Watson-Crick (stars) or the Wobble (plus) base-pairing with EBS motifs were used to construct the consensus homing site. Stem-loops belonging to Rho-independent transcription terminators inferred by ARNold [58] are colored in blue (stems) and red (loops). Underlined sequences are stem-loops not detected by ARNold. (**C**) Weblogo diagram of the −25 to +10 region surrounding the 26 full-length c-*Ha.de*.I1 copies belonging to subgroup 1. Bases which show relative conservation were used to construct the consensus homing site.

Homing site occupation can also be estimated for potentially active copies of *Kl.pn*.I5 (in *Klebsiella pneumoniae* MGH), *Di.ze*.I1 (in *Dickeya zeae* Ech1591), *So.gl*.I1 (in *Sodalis glossinidius* 'morsitans'), and c-*Ha.de*.I1 (in *Hamiltonella defensa* 5AT). The two *Kl.pn*.I5 copies are identical and inserted in intergenic regions harboring well conserved IBS sequences, and comparison with the −25 to +10 flanking sequences of the reference *Kl.pn*.I5 (located in the *K. pneumoniae* plasmid pK245, *cf.* Zbase) extends the putative homing site to some additional bases (Figure 3A). When searching the MGH genome for available homing sites, no occurrence was detected, indicating an homing site saturation (Table 2). *Di.ze*.I1 and *So.gl*.I1 are bacterial class C introns and detected copies are located downstream of Rho-independent transcription terminators, as expected (Figure 3B). Target regions also show well conserved IBS1 and IBS3 motifs, indicating that these detected copies resulted from retrohoming. The search for Rho-independent transcription terminators followed by consensus IBS1/3 sequences in *D. zeae* and *S. glossinidius* genomes yielded a homing site occupation of 11% (2/17) and 4% (3/73) for *Di.ze*.I1 and *So.gl*.I1 introns, respectively (Table 2).

c-*Ha.de*.I1 in *H. defensa* is represented by 29 full-length copies, for which a previous analysis reported an average nucleotide divergence <2% [68]. Our inspection of c-*Ha.de*.I1 sequences revealed that the pool of copies is distributed into two sub-groups separated by 6% average divergence. Subgroup 1 includes 26 copies showing an average nucleotide divergence of 0.1%. Subgroup 2 encompasses two identical copies and a third one with 2.5% divergence, largely due to a ∼700 bp-long gene conversion event (involving a subgroup 1 copy) which artificially inflates the genetic distance with the two other subgroup 2 copies (Figure S1). The two subgroups thus probably result from two recent, independent proliferation events. Inspection of regions surrounding insertion sites revealed that *c-Ha.de*.I1 copies from subgroup 2 are all inserted in ISPlu15-like IS elements at a specific position. No intron-free occurrence of this IS was found in the chromosomal genome, but one degraded fragment including the homing site was detected in the plasmid (Table 2). By contrast, copies from subgroup 1 show a variety of genomic insertion sites. For example, one copy is inserted in another type of ISPlu15-like IS element (at the same position though), another copy is inserted in an IS427-like element, and three copies are inserted in other copies of *c-Ha.de*.I1 (at a specific position). Some conserved positions can however be detected within −25 to +15 intron flanking sequences, which were used to infer a consensus homing site (Figure 3C). According to this consensus, 17 homing sites (excluding uninterrupted intron copies) are still available in the *H. defensa* genome analyzed here (Table 2). Thus, c-*Ha.de*.I1 subgroup 1 occupies only 60% of its potential homing sites in *H. defensa*.

Overall, among the 16 cases in which proliferation of a potentially active group II intron can be estimated, only 5 exhibit a homing site occupation larger than 80%, and in all cases the number of potential homing sites was lower than 10. By contrast, none of the five genomes which harbored more than 10 potential homing sites for recently acquired group II introns are saturated. These results mainly support the Sel-DE model, in which genomes overloaded by group II intron copies are rapidly eliminated from bacterial populations.

### Fate of Past Group II Intron Acquisitions and Proliferations

The section above provided homing site occupation for recently acquired and still potentially active group II introns. The fate of these proliferations on the long term is a critical difference between the Sat-DE and Sel-DE models (Figure 1). The Sat-DE model predicts that ancient proliferations may be visible for a while in bacterial genomes as degraded copies spanning all potential homing sites. On the contrary, the Sel-DE model predicts that ancient proliferations should not be observable because of the selection pressure acting on intron-loaded genomes. The Sel-DE model also predicts that intron copies may sometimes escape the selection filter when they are inactivated by mutation before proliferation.

One hundred eleven group II intron fragments were detected in our analysis, distributed in 55 distinct copies (Table 1). Most of them are only distantly related to a described active group II intron (Table S2), and their potential homing site cannot be inferred. We thus restricted our analysis to group II intron fragments related to full-length elements described in the previous section and from which we have information on putative homing sites. Two distinct *E.c*.I9 fragments were detected in 7 and 3 *E. coli* genomes, respectively. All these genomes also harbor several free Rsh IS copies, leading to a maximum homing site occupation of 33% (Table 3). Four distinct *E.c*.I3 fragments were detected, one on a *E. coli* E24377A plasmid and all others located on the *Shigella* virulence plasmid. Again, putative available homing sites were detected in host genomes, with 6 occurrences in *E. coli* E24377A and one in each *S. boydii* genome (Table 3). Four distinct *E.c*.I4 fragments were also detected in the *Escherichia/Shigella* genus. Putative homing site occupation is ≤50% in all *E. coli* strains, and <10% in all *Shigella* strains (Table 3). However, similar to inactivated full-length *E.c*.I6, all *E.c*.I4 fragments are located in large MGEs (Table S2), and we cannot exclude a scenario in which they have been acquired already inactivated and unable to proliferate. Two fragments of *So.gl.I1* were detected in the *S. glossinidius* genome. When these copies were inactivated (that we assume before more recent copies were acquired), 76 homing sites still remained available (Table 3). Finally, four fragments of *c-Ha.de.I1* were detected in *the H. defensa* genome, although three of them show 100% identity with copies from subgroup 1 and may not result from past proliferations. The last one is also related to subgroup 1 copies, but with 4% divergence. If we assume that its homing site was the same as those of subgroup 1 copies, this fragment left at least 98% (48/49) of its potential homing sites free before being inactivated (Table 3).

**Table 3:** Group II intron fragments, number of potential homing sites available in their host genomes, and proportion of homing site occupation.

Group II intron	Host genome	Fragmentnumber	Availablehoming sites*	Proportion ofoccupation (%)
*E.c*.I9	*E. coli* BL21	1	4	20
	*E. coli* BL21_DE3	1	4	20
	*E. coli* REL606	1	5	17
	*E. coli* IAI1	1	3	25
	*E. coli* SE11	1	6	14
	*E. coli* E24377A	1	3	25
	*E. coli* EDL933	1	2	33
	*E. coli* Sakai	1	2	33
	*E. coli* EC4115	1	2	33
	*E. coli* TW14359	1	2	33
*E.c*.I3	*E. coli* E24377A	1	6	14
	*S. sonnei* Ss046	3	0	100
	*S. boydii* Sb227	3	1	75
	*S. boydii* SbCDC3083	3	1	75
	*S. flexneri* Sf301	3	0	100
	*S. dysenteriae* Sd197	3	0	100
*E.c*.I4	*E. coli* BW2952	1	1	50
	*E. coli* DH10B	1	1	50
	*E. coli* MG1655	1	1	50
	*E. coli* W3110	1	4	50
	*E. coli* SMS_3_5	1	10	9
	*E. coli* UTI89	1	3	25
	*E. coli* UMN026	1	7	13
	*S. sonnei* Ss046	1	15	6
	*S. boydiii* SbCDC3083	1	57	2
	*S. flexneri* Sf301	1	43	2
	*S. dysenteriae* Sd197	1	25	4
*So.gl*.I1	*S. glossinidius* 'morsitans'	2	76	3
*c-Ha.de*.I1 (subgroup 1)	*H. defensa* 5AT	1	49	2

*: at the expected time of fragment acquisition (*i.e.* including homing sites currently occupied by full-length copies).

In summary, despite numerous group II intron fragments detected in our dataset, no evidence of any large ancient proliferation that led to homing site saturation can be identified. On the contrary, detected fragments generally show no counterpart belonging to the same intron in the same genome, despite the presence of putative available homing sites. This suggests that these copies were inactivated before their proliferation, or that genomes in which proliferation occurred didn't survive the purifying selection filter. All these observations are more consistent with a Sel-DE model than with a Sat-DE model for explaining group II intron dynamics.

## Discussion

Group II intron distribution in bacteria is known to be highly variable in abundance and diversity, suggesting periodic extinction-recolonization events [41]–[43]. However, whether extinctions are driven by purifying selection acting on group II intron proliferation (Sel-DE model) or by slow degradation of elements after genome saturation (Sat-DE model) was still unclear. Our analysis of group II introns in *Enterobacteriales* first confirms their heterogeneous distribution between and within bacterial species, and their tendency to be located in larger MGEs which may favor their horizontal transfer, consistent with an extinction-recolonization model. Next, introns subject to recent (or ongoing) and past proliferation events have not reached homing site saturation in genomes they colonized, except when the number of homing sites was very low. The Sat-De model assumes that there is no other barrier to proliferation than the number of available homing sites when a new group II intron enters a genome. On the contrary, the Sel-DE model assumes that the more loaded with group II introns copies the genomes are, the more they are counter-selected, even if not all homing sites are filled. Our observations thus better support group II intron dynamics constrained by selective forces limiting intra-genomic proliferations.

Deleteriousness of inserted elements is a prerequisite of the Sel-DE model, because it conditions their quick removal from the bacterial populations [17], [21]. Individual TE copies may be deleterious when they insert within genes or regulatory regions [5], [69]. In principle, group II introns inserted in transcribed regions have the ability to precisely self-splice from the RNA transcript, thereby allowing the correct expression of the genomic region they are inserted in [29]. Moreover, this study and others show that group II introns are generally located within MGEs or other non essential genomic regions [26], [42], [45], [70]. Sel-DE dynamics for these elements may therefore be somewhat unexpected, as intron insertions would be expected to have no or limited effect on bacterial fitness. Two explanations can resolve this apparent contradiction. First, group II introns may be found only in non-essential genomic regions because copies inserted in essential genes are so deleterious that they are quickly removed from the population and virtually undetectable. This would imply that the splicing efficiency of group II introns should be called into question, or at least their efficiency to render viable exons after splicing. Consistently, it was observed that introns located in an IS element and in a gene only essential for plasmid propagation were respectively very and moderately inefficient for reconstructing the interrupted exon after splicing [49], [50]. On the other hand, an active group II intron was detected in the essential *recA* gene of *Geobacillus kaustophilus*, in which it seems not to alter host survival [71]. This intron shows hallmarks of host adaptation (based on amelioration of nucleotide composition), and it may also have increased its exon-ligation efficiency to lower its impact on host fitness. The question of exon-ligation efficiency after splicing is thus still in great debate and clearly needs further experimental investigation.

Alternatively, group II intron deleterious effect may be caused by the accumulation of copies rather than by the deleteriousness of individual insertions. Repetitive elements, particularly TEs, are known to be factors of genomic instability because they are preferred targets of ectopic homologous recombination events [69], [72], [73], and this holds true for group II introns as well [43]. Although TE-mediated genomic rearrangements may occasionally result in genetic innovations [5], [74], most events are structurally deleterious and subject to purifying selection. A large number of homologous TEs within a genome is thus by nature counter-selected, as it mechanistically increases the probability of genomic rearrangements. Increasing the number of active copies also increases the overall activity of a given element. For group II introns, it would result in an accumulation of RNP particles wandering for available homing sites, increasing as much the probability of retrotransposition (non-specific insertions), sometimes in important genes.

The impact of negative selection on group II intron proliferation probably shaped the diversity of homing site targeting strategies. For instance, bacterial class C introns target downstream regions of transcription terminators, which means that they are never transcribed except when the transcription machinery miss the end checkpoint. This kind of specific targeting can thus be viewed as a self-regulation strategy to limit intron proliferation [26], [38]. Our data perfectly illustrate such a self-regulation process, with a very low number of *Di.ze*.I1 and *So.gl*.I1 detected in *D. zeae* and *S. glossinidius* genomes, respectively, despite numerous potential homing sites (Table 2). Another strategy widely used by group II introns is to target IS elements [40], [44], [46]. This homing strategy provides several advantages, such as numerous putative homing sites, located in non essential genes, and which may help for horizontal transfers. Moreover, IS element abundance itself is limited by selection [17], which means that intron proliferations are *de facto* controlled. Surprisingly, among the nine *Escherichia/Shigella* genomes harboring the IS-targeting *E.c*.I4, only three show intron saturation (Table 2). Although it may be caused by intron inactivity (at least for genomes with only one full-length representative), it may also result from a cumulative effect of repeated elements on genome instability. Indeed, intron/IS tandems span >3 kb in length, while IS alone are generally ∼1 kb-long. As recombination frequency increases with the size and similarity of the homologous region [75], [76], IS interrupted by group II introns may be more prone than IS or introns alone to induce genomic rearrangements. Thus, group II intron proliferation within other repeated elements (such as IS) may be even more efficiently counter-selected than proliferation into less specific target regions.

Bacterial lifestyle may also play a critical role in group II intron proliferation. Reduction of effective population size of bacteria is generally linked with severe proliferations of IS elements, because of a weakening of the efficiency of purifying selection [77]–[79]. This process is particularly striking in intracellular bacteria: genomes of facultative intracellular or recent obligate endosymbionts tend to exhibit larger IS abundance compared to those of free-living species, which generally contain very few IS elements [63], [77]. Interestingly, two of the facultative endosymbionts in our dataset (*H. defensa* and *S. glossinidius*), have experienced recent group II intron proliferations. However, reduction of the efficiency of purifying selection only allows the proliferation of elements that were already present and functional in the genome of these bacteria. This is fairly well illustrated by the facultative intracellular bacterium *Y. pestis*, in which all IS families that have proliferated were present in the free-living ancestor *Y. pseudotuberculosis* [80]. Thus, at least one active group II intron should have been present in the genome of bacteria that shifted toward intracellularity to benefit from the reduction of effective population size and proliferate. This may explain why the three young and facultative intracellular species *P. asymbiotica*, *S flexneri*, and *Y. pestis* do not show any evidence for recent group II intron proliferation.

Intracellularity may also have a further impact on Sel-DE dynamics: such bacteria are confined within host cells, which reduces the opportunities for MGE exchange with other bacteria. For example, obligate mutualistic bacteria, which are transmitted essentially vertically and are clustered within specific cellular compartments, show virtually no DNA acquisition [63]. As these bacteria no longer exchange plasmids or phages, they are not subject to the first phase of the Sel-DE model, and are therefore “protected” against new group II intron colonizations. In our dataset, nine genomes are from obligate mutualistic endosymbionts (six *Buchnera aphidicola* strains, two *candidatus Blochmania spp.* strains, and one *Wiggleworthia glossinidia* strain). As expected, no group II intron was detected in their genomes, consistent with the view that they have eliminated copies of pre-mutualistic stage proliferations, and that their strict intracellular confinement mainly protects them against new invasions.

Group I intron dynamics in organelles follow a saturation-driven extinction (Sat-DE) model, and it was proposed that this model could fit to other target-primed retroelements in other organisms [25]. On the contrary, our study indicates that group II introns evolve under a selection-driven extinction (Sel-DE) model initially proposed to describe the evolution of IS elements in bacterial genomes [17]. It emphasizes that Sel-DE dynamics are mostly constrained by life history traits of their bacterial hosts rather than by TE intrinsic properties, although TE characteristics (such as specific or non-specific target insertion sites) may play a role on their relative abundance within genomes. This could explain why IS elements are generally much more common than group II introns in bacterial genomes. Effective population size as well as lateral gene transfers have also critical effects on these dynamics. Most bacteria show two of the prerequisites for Sel-DE dynamics, *i.e.* large population sizes and frequent genetic exchanges, contrary to eukaryotes which generally show smaller effective population sizes and lower rates of lateral gene transfer, and for which other TE evolutionary strategies were observed [74]. Yet, the Sel-DE model may also apply to some extent to eukaryote TEs such as *mariner* elements, which experience frequent horizontal transfers in their life cycles, followed by rapid proliferation and degeneration in host genomes [81], [82]. Whether Sel-DE dynamics apply to other eukaryote TEs is an open and essential question, which could be investigated for example by looking at TE dynamics in eukaryotes sharing properties similar to those of bacteria (e.g. unicellular free living eukaryotes).

## Supporting Information

Figure S1 **Sequence alignment of the three c-** ***Ha.de*** **.I1 copies belonging to subgroup 2 and one representative copy of subgroup 1.** Bases identical to the subgroup 2 reference (c-*Ha.de*.I1-10) are dotted. Stars denote start and end points of the converted region in c-*Ha.de*.I1-1.
(TIFF)

Table S1 **Genome information and group II intron content for the 90 analyzed** ***Enterobacteriales*** **strains.**
(PDF)

Table S2 **List of the 135 distinct group II intron copies found in the 90** ***Enterobacteriales*** **strains, with detailed information.** ***E.c*** **.I9-1/tr1 and** ***E.c*** **.I5-1/tr1 are full-length in some** ***Escherichia coli*** **strains and fragmented in others; they are counted only as distinct full-length elements.** Attributed intron names are those of the most closely related group II introns in Zbase when the nucleotide similarity is >90%, and new names otherwise (see “*Notes*” column).
(PDF)

Table S3 **Genomic start and end positions of the 198 detected group II intron copies, listed by genus. Fragment positions of interrupted copies are separated with slashes.**
(PDF)
