# Supplementary figures and images for: Selection-Driven Extinction Dynamics for Group II Introns in Enterobacteriales
Source: PLoS One. 2012 Dec 14;7(12):e52268. doi: 10.1371/journal.pone.0052268 (PMC3522654; doi:10.1371/journal.pone.0052268)

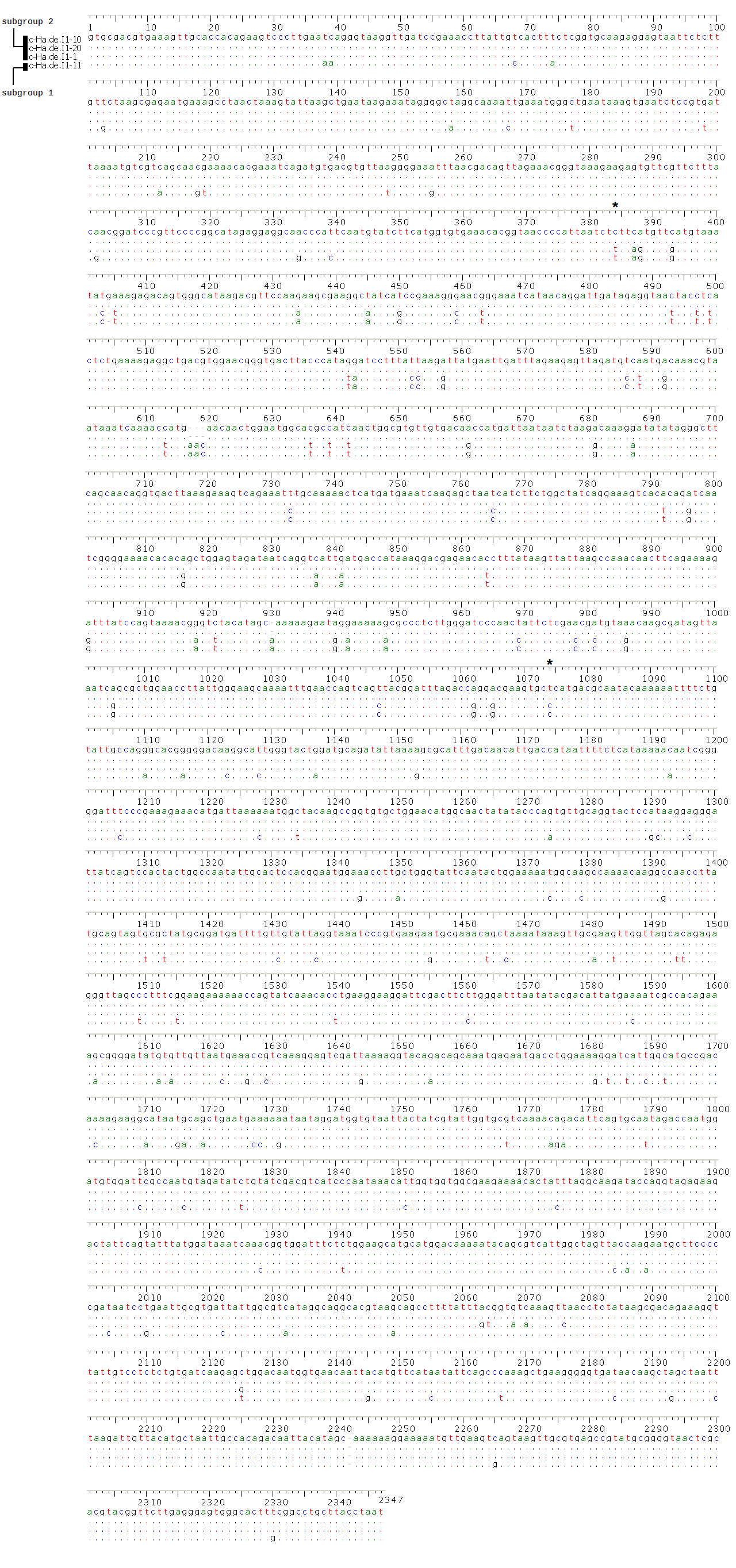

Supplement: Figure S1 — Sequence alignment of the three c- Ha.de .I1 copies belonging to subgroup 2 and one representative copy of subgroup 1. Bases identical to the subgroup 2 reference (c-Ha.de.I1-10) are dotted. Stars denote start and end points of the converted region in c-Ha.de.I1-1. (TIFF) [file pone.0052268.s001.tif]
