# Supplementary material for: Selection-Driven Extinction Dynamics for Group II Introns in Enterobacteriales
Source: PLoS One. 2012 Dec 14;7(12):e52268. doi: 10.1371/journal.pone.0052268 (PMC3522654; doi:10.1371/journal.pone.0052268)
Supplement: Table S1 — Genome information and group II intron content for the 90 analyzed Enterobacteriales strains. (PDF) [file pone.0052268.s002.pdf]

List of the 90 Enterobacteriale sequenced strains, with their full-length and fragmented intron abundance in plasmids and chromosomes

genus	group	strain	GenBank accession	number of free plasmids	group II introns in plasmids full-length	group II introns in plasmids fragments	group II introns in chromosome full-length	group II introns in chromosome fragments	**total**	strain-specific copies
Escherichia/Shigella										
	**B2**	**E. coli O127H6 str. E2348/69**	NC_011601	2	.	.	.	.		.
		**E. coli 536**	NC_008253		.	.	1	.	**1**	1
		**E. coli CFT073**	NC_004431		.	.	1	1	**2**	1
		**E. coli ED1a**	NC_011745		.	.	5	.	**5**	3
		**E. coli S88**	NC_011742	1	.	.	1	1	**2**	
		**E. coli APEC_O1**	NC_008563	2	.	.	1	1	**2**	
		**E. coli UTI89**	NC_007946	1	*1*	*1*	.	.	**2**	
	**D**	**E. coli IAI39**	NC_011750		.	.	15	.	**15**	15
		**E. coli SMS-3-5**	NC_010498	4	.	.	1	2	**3**	3
		**E. coli UMN026**	NC_011751	2	*1*	*1*	1	.	**3**	1
	**A**	**E. coli ATCC 8739**	NC_010468		.	.	1	.	**1**	
		**E. coli HS**	NC_009800		.	.	.	.		.
		**E. coli BL21**	NC_012892		.	.	.	1	**1**	
		**E. coli BL21-Gold (DE3)**	NC_012947		.	.	.	1	**1**	
		**E. coli B str. REL606**	NC_012967		.	.	.	1	**1**	
		**E. coli K12 BW2952**	NC_012759		.	.	.	1	**1**	
		**E. coli K12 substr. DH10B**	NC_010473		.	.	.	1	**1**	
		**E. coli K12 substr. MG1655**	NC_000913		.	.	.	1	**1**	
		**E. coli K12 substr. W3110**	AC_000091		.	.	.	1	**1**	
	**B1**	**E. coli IAI1**	NC_011741		.	.	.	2	**2**	
		**E. coli SE11**	NC_011415	6	.	*2*	.	2	**4**	2
		**E. coli 55989**	NC_011748		.	.	6	1	**7**	5
		**E. coli E24377A**	NC_009801	6	*4*	*2*	3	3	**12**	9
	**SS**	**S. sonnei Ss046**	NC_007384	4	.	*8*	.	1	**9**	
		**S. boydii Sb227**	NC_007613	1	.	*5*	.	1	**6**	
		**S. boydii CDC 3083-94**	NC_010658	5	.	*10*	.	1	**11**	1
	**S3**	**S. flexneri Sf_5a_8401**	NC_008258		.	.	.	1	**1**	
		**S. flexneri 2a str. 301**	NC_004337	1	.	*10*	1	1	**12**	2
		**S. flexneri 2a 2457T**	NC_004741		.	.	1	1	**2**	
	**S.D.**	**S. dysenteriae Sd197**	NC_007606	2	.	*6*	.	.	**6**	
	**E**	**E. coli O157H7 str. EDL933**	NC_002655	1	*1*	.	.	1	**2**	
		**E. coli O157H7 str. Sakai**	NC_002695	2	*1*	.	.	1	**2**	
		**E. coli O157H7 str. EC4115**	NC_011353	2	.	.	.	1	**1**	
		**E. coli O157H7 str. TW14359**	NC_013008	1	.	.	.	1	**1**	
		E. fergusonii ATCC 35469	NC_011740	1	.	*1*	.	.	**1**	1
		**genus average/sub-TOTAL**		**1.26**	***8***	***46***	**38**	**30**	**122**	**44**
		sub-TOTAL for distinct copies			*6*	*20*	34	7	67	44
Yersinia										
		Y. pestis Angola	NC_010159	2	.	*1*	.	.	**1**	
		Y. pestis Antiqua	NC_008150	3	.	*1*	.	.	**1**	
		Y. pestis Microtus str. 91001	NC_005810	4	.	*1*	.	.	**1**	
		Y. pestis CO92	NC_003143	3	.	*1*	.	.	**1**	
		Y. pestis KIM 10	NC_004088	1	.	*1*	.	.	**1**	
		Y. pestis Nepal516	NC_008149	2	.	*1*	.	.	**1**	
		Y. pestis Pestoides F	NC_009381	2	.	*1*	.	.	**1**	
		Y. enterocolitica 8081	NC_008800	1	.	*1*	.	.	**1**	1
		Y. pseudotuberculosis IP 31768	NC_009708	2	.	.	.	.		.
		Y. pseudotuberculosis IP 32953	NC_006155	2	.	.	.	.		.
		Y. pseudotuberculosis PB1/+	NC_010634	1	.	.	.	.		.
		Y. pseudotuberculosis YPIII	NC_010465		.	.	.	.		.
		**genus average/sub-TOTAL**		**1.92**		***8***			**8**	**1**
		sub-TOTAL for distinct copies				*2*			2	1
Klebsiella										
		K. pneumoniae 342	NC_011283	2	.	.	.	.		.
		K. pneumoniae MGH 78578	NC_009648	5	*1*	*1*	1	1	**4**	4
		K. pneumoniae NTUH-K2044	NC_012731	1	.	.	.	1	**1**	1
		**genus average/sub-TOTAL**		**2.67**	***1***	***1***	**1**	**2**	**5**	**5**
		sub-TOTAL for distinct copies			*1*	*1*	1	2	5	5
Dickeya										
		D. dadantii Ech703	NC_012880		.	.	1	.	**1**	
		D. zeae Ech1591	NC_012912		.	.	3	.	**3**	2
		**genus average/sub-TOTAL**					**4**		**4**	**2**
		sub-TOTAL for distinct copies					3		3	2
Photorabdus										
		P. luminescens TTO1	NC_005126		.	.	2	7	**9**	8
		P. asymbiotica ATCC 43949	NC_012962	1	.	.	.	1	**1**	
		**genus average/sub-TOTAL**		**0.5**			**2**	**8**	**10**	**8**
		sub-TOTAL for distinct copies					2	7	9	8

Sodalis									
	S. glossinidius 'morsitans'	NC_007712	3	.	*1*	3	4	**8**	8
	**genus average/sub-TOTAL**		**3**		***1***	**3**	**4**	**8**	**8**
	sub-TOTAL for distinct copies				***1***	**3**	**4**	**8**	**8**
Serratia									
	S. proteamaculans 568	NC_009832	1	.	.		3	**3**	3
	**genus average/sub-TOTAL**		**1**				**3**	**3**	**3**
	sub-TOTAL for distinct copies						**3**	**3**	**3**
Candidatus Hamiltonella									
	H. defensa 5AT	NC_012751	1	*1*	.	29	8	**38**	38
	**genus average/sub-TOTAL**		**1**	***1***		**29**	**8**	**38**	**38**
	sub-TOTAL for distinct copies			***1***		**29**	**8**	**38**	**38**
Buchnera									
	**B. aphidicola Bp**	NC_004545	1	.	.	.	.		.
	**B. aphidicola 5A**	NC_011833		.	.	.	.		.
	**B. aphidicola Cc**	NC_008513	1	.	.	.	.		.
	**B. aphidicola Sg**	NC_004061		.	.	.	.		.
	**B. aphidicola Tuc7**	NC_011834		.	.	.	.		.
	**B. aphidicola APS**	NC_002528	2	.	.	.	.		.
	**genus average/sub-TOTAL**		**0.67**						
	sub-TOTAL for distinct copies								
Candidatus blochmannia									
	B. floridanus	NC_005061		.	.	.	.		.
	B. pennsylvanicus BPEN	NC_007292		.	.	.	.		.
	**genus average/sub-TOTAL**								
	sub-TOTAL for distinct copies								
Wigglesworthia									
	W. glossinidia	NC_004344	1	.	.	.	.		.
	**genus average/sub-TOTAL**		**1**						
	sub-TOTAL for distinct copies								
Salmonella									
	**S. enterica arizonae RSK2980**	NC_010067		.	.	.	.		.
	**S. enterica Choleraesuis SC-B67**	NC_006905	2	.	.	.	.		.
	**S. enterica Schwarzengrund CVM1**	NC_011094	2	.	.	.	.		.
	**S. enterica Agona SL483**	NC_011149	1	.	.	.	.		.
	**S. enterica Dublin CT_02021853**	NC_011205	1	.	.	.	.		.
	**S. enterica Enteritidis P125109**	NC_011294		.	.	.	.		.
	**S. enterica Gallinarum 287/91**	NC_011274		.	.	.	.		.
	**S. enterica Heidelberg SL476**	NC_011083	2	.	.	.	.		.
	**S. enterica Newport SL254**	NC_011080	2	.	.	.	.		.
	**S. enterica Paratyphi A ATCC 915**	NC_006511		.	.	.	.		.
	**S. enterica Paratyphi A AKU_1260**	NC_011147		.	.	.	.		.
	**S. enterica Paratyphi B SPB7**	NC_010102		.	.	.	.		.
	**S. enterica Paratyphi C RKS4594**	NC_012125	1	.	.	.	.		.
	**S. enterica Typhi Ty2**	NC_004631		.	.	.	.		.
	**S. enterica Typhi CT18**	NC_003198	2	.	.	.	.		.
	**S. enterica Typhimurium LT2**	NC_003197	1	.	.	.	.		.
	**genus average/sub-TOTAL**		**0.875**						
	sub-TOTAL for distinct copies								
Citrobacter									
	C. koseri ATCC BAA-895	NC_009792	2	.	.	.	.		.
	**genus average/sub-TOTAL**		**2**						
	sub-TOTAL for distinct copies								
Proteus									
	P. mirabilis HI4320	NC_010554	1	.	.	.	.		.
	**genus average/sub-TOTAL**		**1**						
	sub-TOTAL for distinct copies								

Edwardsiella									
	E. ictaluri 93-146	NC_012779		.	.	.	.		.
	**genus average/sub-TOTAL**								
	sub-TOTAL for distinct copies								
Enterobacter									
	Enterobacter sp 638	NC_009436	1	.	.	.	.		.
	**genus average/sub-TOTAL**		**1**						
	sub-TOTAL for distinct copies								
Cronobacter									
	C. sakazakii ATCC BAA-894	NC_009778	2	.	.	.	.		.
	**genus average/sub-TOTAL**		**2**						
	sub-TOTAL for distinct copies								
Pectobacterium/Erwinia									
	E. tasmaniensis Et1/99	NC_010694	5	.	.	.	.		.
	P. atroscepticum SCRI1043	NC_004547		.	.	.	.		.
	P. carotovorum PC1	NC_012917		.	.	.	.		.
	**genus average/sub-TOTAL**		**1.67**						
	sub-TOTAL for distinct copies								
	**TOTAL**		***1.20***	***10***	***56***	**77**	**55**	**198**	**109**
	for distinct copies			***8***	***24***	**72**	**31**	**135**	**109**

number of genomes with zero intron : 40
