# Supplementary material for: Selection-Driven Extinction Dynamics for Group II Introns in Enterobacteriales
Source: PLoS One. 2012 Dec 14;7(12):e52268. doi: 10.1371/journal.pone.0052268 (PMC3522654; doi:10.1371/journal.pone.0052268)
Supplement: Table S2 — List of the 135 distinct group II intron copies found in the 90 Enterobacteriales strains, with detailed information. E.c .I9-1/tr1 and E.c .I5-1/tr1 are full-length in some Escherichia coli strains and fragmented in others; they are counted only as distinct full-length elements. Attributed intron names are those of the most closely related group II introns in Zbase when the nucleotide similarity is >90%, and new names otherwise (see “Notes” column). (PDF) [file pone.0052268.s003.pdf]

List of the 136 distinct group II intron copies, and their orthology relationships

genus	Locus	Full-length	fragment	strain specific	evidence of retrohoming	number of orthologs	Located in a : free plasmid	integrated MGE (no IS)	Host genome(s)	class	size	Notes
Escherichia/Shigella												
	E.c.I9-1/tr1	X	X			8			ATCC_8739, IAI1, SE11, E24377A, EDL933, Sakai, EC4115,TW14359	D		In a Rhs region. One full-length copy in ATCC_8739, the seven other orthologues are fragments of 193 bp
	E.c.I9-tr2		X			3			BL21, BL21_DE3, REL606	D		In another Rhs region. Fragment of 159 bp
	E.c.I2-1	X		X		1			UMN026	D		In a Rhs region.
	E.c.I3-1	X		X		1		X	ED1a	D		strain-specific GI
	E.c.I3-2a	X		X		1		X	ED1a	D		strain-specific PAI1
	E.c.I3-2b	X		X		1		X	536	D		strain-specific PAI1, inserted at a different locus compared to ED1a
	E.c.I3-tr1		X	X		1	X		E24377A	D		1419 bp fragment 96-98% similar to E.c.I-1. With internal deletion and IS insertion.
	E.c.I3-tr2		X			5	X		Sf301, SbCDC3083, Ss046, S. dysenteriae, Sb227	D		1789 bp fragment 98% similar to the E. coli E.c.I3-1. Lacks 37 bp and 900 bp of the host IS679 in 5'. 1295 bp on Sb227 + interrupted by an IS
	E.c.I3-tr3		X			5	X		Sf301, SbCDC3083, Ss046, S. dysenteriae, Sb227	D		120/122 bp fragment 95% similar to the E. coli E.c.I3-1. Same plasmid than E.c.I3-tr2
	E.c.I3-tr4		X			3	X		Sf301, SbCDC3083, Ss046	D		360-619 bp fragment 95% similar to the E. coli E.c.I3-1. Same plasmid than E.c.I3-tr2. Grouped in Zbase with S.s.F2
	E.c.I4-1	X		X		1		X	ED1a	A		Strain-specific integrated plasmid
	E.c.I4-2a	X		X	X	1		X	ED1a	A		Evidence of retrohoming in prophage P4 (MAGE comparison with S88)
	E.c.I4-2b	X				2		X	E24377A, 55989	A		Inserted at the same locus than in ED1a
	E.c.I4-3a	X		X		1		X	55989	A		strain-specific SHI-1, the IS host is a chimeric IS629/IS911
	E.c.I4-3b	X				2		X	Sf301, Sf2457T	A		In SH1-1 inserted at a different locus compared to 55989
	E.c.I4-4	X		X		1		X	55989	A		In the iuc/iut 62 kbp strain-specific GI
	E.c.I4-5	X		X		1		X	55989	A		same strain-specific GI than E.c.I4-4
	E.c.I4-6	X		X		1		X	55989	A		same strain-specific GI than E.c.I4-4
	E.c.I4-7	X		X		1		X	55989	A		same strain-specific GI than E.c.I4-4
	E.c.I4-8	X		X	X	1		X	E24377A	A		Evidence of retrohoming (MAGE comparison with 55989)
	E.c.I4-9	X		X		1		X	E24377A	A		Strain-specific GI
	E.c.I4-10	X		X		1			IAI39	A		Strain-specific IS
	E.c.I4-11	X		X		1		X	IAI39	A		Strain-specific GI
	E.c.I4-12	X		X		1		X	IAI39	A		Strain-specific prohage
	E.c.I4-13	X		X		1		X	IAI39	A		Strain-specific prohage
	E.c.I4-14	X		X		1		X	IAI39	A		Strain-specific prohage
	E.c.I4-15	X		X		1		X	IAI39	A		same prophage than E.c.I4-14. Host IS is a chimeric IS629/ISEc31
	E.c.I4-16	X		X		1			IAI39	A		Strain-specific IS
	E.c.I4-17	X		X		1			IAI39	A		Strain-specific IS
	E.c.I4-18	X		X		1		X	IAI39	A		Strain-specific 33 kbp prophage
	E.c.I4-19	X		X		1			IAI39	A		Strain-specific IS
	E.c.I4-20	X		X		1			IAI39	A		Strain-specific IS
	E.c.I4-21	X		X		1			IAI39	A		Strain-specific IS
	E.c.I4-22	X		X		1			IAI39	A		degraded IS
	E.c.I4-23	X		X		1			IAI39	A		Strain-specific IS
	E.c.I4-24	X		X		1		X	IAI39	A		Strain-specific 50 kbp prophage
	E.c.I4-25	X		X		1		X	SMS-3-5	A		Strain-specific GI (cf SMS-3-5 publication)
	E.c.I4-26	X				2	X		UTI89, UMN026	A		
	E.c.I4-27	X		X		1	X		E24377A	A		
	E.c.I4-28	X		X		1	X		E24377A	A		in the same plasmid than E.c.I4-27
	E.c.I4-29	X		X		1	X		E24377A	A		in the same plasmid than E.c.I3-tr1
	E.c.I4-30	X		X		1	X		E24377A	A		
	E.c.I4-tr1		X			4		X	DH10B, BW2952, MG1655, W3110	A		prophage CP4-6. Fragment of 1764 bp
	E.c.I4-tr2		X	X		1		X	SMS-3-5	A		same strain-specific GI than E.c.I4-25. Fragment of 132 bp.
	E.c.I4-tr3		X			2	X		UTI89, UMN026	A		in the same plasmid than E.c.I4-26. Fragment of 985 bp
	E.c.I4-tr4		X			4	X		Sf301, SbCDC3083, Ss046, S. dysenteriae	A		Fragment of 405 bp. Same plasmid than E.c.I3-tr2
	E.c.I5-1/tr1	X	X			3	X		EDL933, Sakai, SbCDC3083	CL1		shared plasmid between the two O157:H7 strains, orthologous but truncated (577 bp) in the plasmid of Shigella boydii CDC3083
	E.c.I6-1a	X				2		X	CFT073, ED1a	C		in PAI SelC-Island
	E.c.I6-1b	X				2		X	APEC_01, S88	C		in the same PAI than E.c.I6-1a, but the PAI is found at a different location
	E.c.I6-tr1		X	X		1			SMS-3-5	C		976 bp fragment, whith an internal deletion. In a degraded region.
	E.c.F10a		X	X		1		X	CFT073	F		177 bp fragment in PAI SelC-Island, 77% similarity with D.a.I1
	E.c.F10b		X			2		X	APEC_01, S88	F		177 bp fragment in PAI SelC-Island. Same fragment than E.c.F10a, but the PAI is located at a different position.
	E.c.F11		X	X		1		X	E24377A	D		1155 bp fragment in Strain-specific 12 kbp GI. 27 % id aa with Sh.dy.I1 ORF
	E.c.F12		X			10			IAI1, SE11, E24377A, 55989, Ss046, Sb227, SbCDC3083, Sf8401, Sf301	C		118 bp fragment with 79% similarity with P.s.I1
	E.c.F13		X	X		1	X		SE11	C		215 bp fragment with 77% similarity with A.v.I2
	E.c.F14		X	X		1	X		SE11	D		500 bp fragment in the same plasmid than E.c.F14. 92% similarity with P.I.I2
	E.c.F15		X	X		1	X		E24377A	C		135 bp fragment with 81% similarity with Di.ze.I1. In the same plasmid than E.c.I3-tr1
	S.s.F1		X			4	X		Sf301, SbCDC3083, Ss046, S. dysenteriae	D		432 bp fragment 90% similar to P.I.I2. Same plasmid than E.c.I3-tr2
	S.s.F2		X			3	X		Sf301, SbCDC3083, Ss046	D		723-1436 bp fragment 89% similar to P.I.I2. Same plasmid than E.c.I3-tr2. Probably the second part of S.s.F1
	S.s.F3		X			2	X		Ss046, S. dysenteriae	D		256 bp fragment 87% similar to P.I.I2. Same plasmid than E.c.I3-tr2
	S.b.F1		X			3	X		Sf301, SbCDC3083, Sb227	CL1		149 bp fragment 79% similar to A.v.I1. Same plasmid than E.c.I3-tr2
	S.b.F2		X			3	X		Sf301, SbCDC3083, Sb227	D		116 bp fragment 76% similar to Ce.ja.I1. Same plasmid than E.c.I3-tr2
	S.b.F3		X			2	X		Sb227, SbCDC3083	D		196 bp fragment 77% similar to Pr.ae.I5. Same plasmid than E.c.I3-tr2
	S.d.F1		X			2	X		Ss046, S. dysenteriae	D		545 bp fragment 82% similar to Di.ze.I1. Same plasmid than E.c.I3-tr2
	S.f.F4		X	X		1	X		Sf301			137 bp fragment 75% similar to Dh.re.I1. Same plasmid than E.c.I3-tr2
	S.f.F5		X	X		1	X		Sf301			135 bp fragment 79% similar to UA.I3. Same plasmid than E.c.I3-tr2
	S.ma.I1-tr1		X	X		1	X		*E. fergusonii*	E1		867 bp fragment 99% similar to S.ma.I1.

		full-length	strict fragments	evidence of retrohoming	plasmidic	in a MGE (free or intergated)
**total**	122	46	76	2	54	91
**distinct**	67	40	27	2	25	54
**Strain-specific**	44	33	11	2	11	45

Yersinia

Y.p.F1			X			7	X		all *Yersinia pestis* strains	A	1143 bp fragment. Corresponds to the Y.p.F1 fragment of Zbase.
Y.e.F1			X	X		1	X		*Yersinia enterolitica*	A	563 bp fragment. Corresponds to the Y.e.F1 fragment of Zbase.

		full-length	fragments	frag on plasmid	evidence of retrohoming	Full-length on plasmid	plasmidic	in a MGE (free or intergated)
**total**	8		8	8			8	8
**distinct**	2		2	2			2	2
**Strain-specific**	1		1	1			1	1

Klebsiella

Kl.pn.F1			X	X		1		X	MGH	C	936 bp fragment in strain-specific 12 kbp GI. 27% aa id with G.s.I1
Kl.pn.F2			X	X		1		X	NTUH	C	1919 bp fragment in strain-specific 60 kbp integrated plasmid. 24% id aa with Ge.sp.I1 ORF
Ps.st.I2-tr1			X	X		1	X		MGH	CL1	220 bp fragment 98% similar to the Ps.st.I2 reference. In the same plasmid than S.ma.I1-2
Kl.pn.I5-1		X		X	X	1			MGH	E1	Evidence of retrohoming (MAGE comparison with NTUH)
Kl.pn.I5-2		X		X		1	X		MGH	E1	

		full-length	fragments	evidence of retrohoming	plasmidic	in a MGE (free or intergated)
**total**	5	2	3	1	2	4
**distinct**	5	2	3	1	2	4
**Strain-specific**	5	2	3	1	2	4

Dickeya

Di.da.I1		X				2			*D.dadantii, D.zeae*	ML	Probably resulting from two independent insertions : 99% identity between the two introns, 90% for the flanking sequence
Di.ze.I1-1		X		X	X	1			*D.zeae*	C	Evidence of retrohoming (MAGE comparison with D. dadantii)
Di.ze.I1-2		X		X	X	1			*D.zeae*	C	Evidence of retrohoming (MAGE comparison with D. dadantii)

		full-length	fragments	evidence of retrohoming	plasmidic	in a MGE (free or intergated)	classes
**total**	4	4		2			C, ML
**distinct**	3	3		2			
**Strain-specific**	2	2		2			

Photorabdus

P.l.I1-1		X		X		1		X	*P. luminescens*	E1	Strain-specific 90 bp GI.
P.l.I1-tr1			X	X		1			*P. luminescens*	E1	1568 bp fragment 98-96% similar to P.l.I1-1.truncated by P.l.I2 and interrupted by an IS
P.l.I1-tr2			X	X		1			*P. luminescens*	E1	171 bp fragment 94% similar to P.l.I1-1
P.l.I1-tr3			X	X		1			*P. luminescens*	E1	244 bp fragment 95% similar to P.l.I1-1
P.l.I2-1		X		X		1			*P. luminescens*	D	
P.l.F1			X			2			*P. luminescens, P. asymbiotica*	ML	181-259 bp fragment 86-88% similar to Di.ze.I2
P.l.F2			X	X		1			*P. luminescens*	ML	325 bp fragment 81% similar to Di.ze.I2
P.l.F3			X	X		1			*P. luminescens*	ML	182 bp fragment 90% similar to Di.ze.I2. 25 bp away from P.l.F2.
P.l.F4			X	X		1			*P. luminescens*	D	135 bp fragment 75% similar to Zu.pr.I1

		full-length	fragments	evidence of retrohoming	plasmidic	in a MGE (free or intergated)	classes
**total**	10	2	8			1	E1, D, ML
**distinct**	9	2	7			1	
**Strain-specific**	8	2	6			1	

Serratia proteomaculans

Se.pr.F1			X	X		1			*S. proteomaculans*	C	978 bp fragment, with a full-length ORF. 26% id aa with Ha.ch.I2 ORF
Se.pr.F2			X	X		1			*S. proteomaculans*	C	196 bp fragment 95% similar to Se.pr.F1
Se.pr.F3			X	X		1			*S. proteomaculans*	C	118 bp fragment 75% similar to Kl.pn.F2

		full-length	fragments	evidence of retrohoming	plasmidic	in a MGE (free or intergated)	classes
**total**	3		3				C
**distinct**	3		3				
**Strain-specific**	3		3				

Sodalis glossinidius

So.gl.I1-1		X		X		1			*S. glossinidius*	C	95-97% similar to So.gl.I1-3. Interrupted by an internal deletion and IS insertion
So.gl.I1-2		X		X		1			*S. glossinidius*	C	99% similar to So.gl.I1-3
So.gl.I1-3		X		X		1			*S. glossinidius*	C	
So.gl.I1-tr1			X	X		1			*S. glossinidius*	C	806 bp fragment 94-96% similar to So.gl.I1-3
So.gl.I1-tr2			X	X		1			*S. glossinidius*	C	487 bp fragment 97% similar to So.gl.I1-3
So.gl.F1			X	X		1			*S. glossinidius*	C	379 bp fragment 87% similar to A.v.I2
So.gl.F2			X	X		1			*S. glossinidius*	C	846 bp fragment 75% similar to A.v.I4. Adjacent to So.gl.tr2
So.gl.F3			X	X		1	X		*S. glossinidius*	E1	279 bp fragment 80% similar to P.l.I1

		full-length	fragments	evidence of retrohoming	plasmidic	in a MGE (free or intergated)	classes
**total**	8	3	5		1	1	C
**distinct**	8	3	5		1	1	
**Strain-specific**	8	3	5		1	1	

Hamiltonella

c-Ha.de.I1-1	X		X	1		X	*Hamilonella defensa*	CL1	All c-Ha.de.I1 have 44-47% id aa with Sm.av.I1 ORF
c-Ha.de.I1-2	X		X	1			*Hamilonella defensa*	CL1	And ≥ 94% similar to each other
c-Ha.de.I1-3	X		X	1			*Hamilonella defensa*	CL1	
c-Ha.de.I1-4	X		X	1			*Hamilonella defensa*	CL1	
c-Ha.de.I1-5	X		X	1			*Hamilonella defensa*	CL1	
c-Ha.de.I1-6	X		X	1			*Hamilonella defensa*	CL1	
c-Ha.de.I1-7	X		X	1			*Hamilonella defensa*	CL1	
c-Ha.de.I1-8	X		X	1			*Hamilonella defensa*	CL1	
c-Ha.de.I1-9	X		X	1		X	*Hamilonella defensa*	CL1	
c-Ha.de.I1-10	X		X	1		X	*Hamilonella defensa*	CL1	
c-Ha.de.I1-11	X		X	1			*Hamilonella defensa*	CL1	
c-Ha.de.I1-12	X		X	1			*Hamilonella defensa*	CL1	
c-Ha.de.I1-13	X		X	1		X	*Hamilonella defensa*	CL1	
c-Ha.de.I1-14	X		X	1			*Hamilonella defensa*	CL1	
c-Ha.de.I1-15	X		X	1			*Hamilonella defensa*	CL1	
c-Ha.de.I1-16	X		X	1			*Hamilonella defensa*	CL1	
c-Ha.de.I1-17	X		X	1			*Hamilonella defensa*	CL1	
c-Ha.de.I1-18	X		X	1			*Hamilonella defensa*	CL1	
c-Ha.de.I1-19	X		X	1			*Hamilonella defensa*	CL1	
c-Ha.de.I1-20	X		X	1		X	*Hamilonella defensa*	CL1	
c-Ha.de.I1-21	X		X	1		X	*Hamilonella defensa*	CL1	
c-Ha.de.I1-22	X		X	1			*Hamilonella defensa*	CL1	
c-Ha.de.I1-23	X		X	1			*Hamilonella defensa*	CL1	
c-Ha.de.I1-24	X		X	1			*Hamilonella defensa*	CL1	
c-Ha.de.I1-25	X		X	1			*Hamilonella defensa*	CL1	
c-Ha.de.I1-26	X		X	1			*Hamilonella defensa*	CL1	
c-Ha.de.I1-27	X		X	1			*Hamilonella defensa*	CL1	
c-Ha.de.I1-28	X		X	1			*Hamilonella defensa*	CL1	
c-Ha.de.I1-29	X		X	1	X		*Hamilonella defensa*	CL1	
c-Ha.de.I1-tr1		X	X	1			*Hamilonella defensa*	CL1	2212 bp fragment 95% similar to the c-Ha.de.I1 reference
c-Ha.de.I1-tr2		X	X	1		X	*Hamilonella defensa*	CL1	129 bp fragment 94% similar to the c-Ha.de.I1 reference
c-Ha.de.I1-tr3		X	X	1			*Hamilonella defensa*	CL1	1869 bp fragment 94% similar to the c-Ha.de.I1 reference
c-Ha.de.I1-tr4		X	X	1			*Hamilonella defensa*	CL1	585 bp fragment 94% similar to the c-Ha.de.I1 reference
c-Ha.de.I2-1	X		X	1			*Hamilonella defensa*	CL1	interrupted by an IS insertion. 50% id aa with E.c.I5 ORF
c-Ha.de.F1		X	X	1			*Hamilonella defensa*	CL1	147 bp fragment 76% similar to Th.e.I8
c-Ha.de.F2		X	X	1			*Hamilonella defensa*	CL1	145 bp fragment 88% similar to c-Ha.de.F1
c-Ha.de.F3		X	X	1			*Hamilonella defensa*	CL1	145 bp fragment 85% similar to c-Ha.de.F1
c-Ha.de.F4		X	X	1			*Hamilonella defensa*	CL1	126 bp fragment 83% similar to E.c.I8

		**full-length**	**fragments**	**evidence of retrohoming**	**plasmidic**	**in a MGE (free or intergated)**	**classes**
**total**	38	30	8		1	8	CL1
**distinct**	38	30	8		1	8	
**Strain-specific**	38	30	8		1	8	

		**full-length**	**fragments**	**evidence of retrohoming**	**plasmidic**	**in a MGE (free or intergated)**	**classes**
**TOTAL**	198	87	111	5	66	113	A,C,D,CL1,ML,E1,F
**distinct**	135	80	55	5	31	70	
**Strain-specific**	109	72	37	5	16	60	
