# Supplementary material for: Selection-Driven Extinction Dynamics for Group II Introns in Enterobacteriales
Source: PLoS One. 2012 Dec 14;7(12):e52268. doi: 10.1371/journal.pone.0052268 (PMC3522654; doi:10.1371/journal.pone.0052268)
Supplement: Table S3 — Genomic start and end positions of the 198 detected group II intron copies, listed by genus. Fragment positions of interrupted copies are separated with slashes. (PDF) [file pone.0052268.s004.pdf]

Table S3

Escherichia/Shigella

Element	Genome	Position	Orientation	Nucleotide Similarity
**E.c.I9-1/tr1**	NC_002695	2050692-2050884	+	97%
	NC_002655	2037589-2037781	-	97%
	NC_009801	1634917-1635109	+	97%
	NC_010468	2411599-2413485	-	100%
	NC_011353	1965283-1965475	+	97%
	NC_011415	1621095-1621287	+	97%
	NC_011741	1525528-1525720	+	97%
	NC_013008	1964259-1964451	+	97%
**E.c.I9-tr2**	NC_012892	242507-242665	+	98%
	NC_012947	3539713-3539871	-	98%
	NC_012967	242510-242668	+	98%
**E.c.I2-1**	NC_011751	269264-271154	+	99%
**E.c.I3-1**	NC_011745	337804-339627	+	97%
**E.c.I3-2a**	NC_011745	4847774-4849597	+	97%
**E.c.I3-2b**	NC_008253	4007952-4009775	-	99%
**E.c.I3-tr1**	NC_009788	27225-28063/ *28296-28746* *30080-30208*	-	98/96/97%
**E.c.I3-tr2**	NC_004851	30084-31910	-	98%
	NC_007608	32965-33803	-	99%
	NC_007607	29397-31222	-	100%
	NC_007385	29758-31491	-	99%
	NC_010660	33803-35629	-	99%
**E.c.I3-tr3**	NC_004851	21636-21755	-	96%
	NC_007608	23915-24036	-	96%
	NC_007607	21615-21734	-	96%
	NC_007385	19693-19812	-	95%
	NC_010660	25890-26009	-	96%
**E.c.I3-tr4**	NC_004851	157225-157686	-	95%
	NC_007608	35856-36311	+	99%
	NC_007385	133641-134259	-	95%
	NC_010660	156731-157090	-	96%
**E.c.I5-1/tr1**	NC_002128	35666-38071	+	100%
	NC_007414	58241-60646	+	100%
	NC_010660	159649-160225	-	99%
**E.c.I6-1a**	NC_004431	4320108-4321914	+	100%
	NC_011745	4320108-4321914	+	100%
**E.c.I6-1b**	NC_008563	4726907-4728713	-	99%
	NC_011742	4641434-4643240	-	99%
**E.c.I6-tr1**	NC_010498	1127307-1127474/ *1127497-1128304*	-	94/98%

Element	Genome	Position	Orientation	Nucleotide Similarity
**E.c.I4-1**	NC_011745	3504972-3507242	-	99%
**E.c.I4-2a**	NC_011745	5081740-5084010	+	99%
**E.c.I4-2b**	NC_009801	4848623-4850893	+	99%
	NC_011748	5044258-5046528	+	99%
**E.c.I4-3a**	NC_011748	3366025-3368295	-	99%
**E.c.I4-3b**	NC_004741	3064669-3066938	-	100%
	NC_004337	3072493-3074763	-	100%
**E.c.I4-4**	NC_011748	4751982-4754252	+	99%
**E.c.I4-5**	NC_011748	4767674-4769944	-	99%
**E.c.I4-6**	NC_011748	4778304-4780574	-	99%
**E.c.I4-7**	NC_011748	4789689-4791959	+	99%
**E.c.I4-8**	NC_009801	3341658-3343928	-	99%
**E.c.I4-9**	NC_009801	4833753-4836023	-	99%
**E.c.I4-10**	NC_011750	489783-492052	-	99%
**E.c.I4-11**	NC_011750	785326-787595	-	99%
**E.c.I4-12**	NC_011750	919953-922222	-	99%
**E.c.I4-13**	NC_011750	1094728-1096997	-	99%
**E.c.I4-14**	NC_011750	1558821-1561090	+	99%
**E.c.I4-15**	NC_011750	1584588-1586857	-	99%
**E.c.I4-16**	NC_011750	1705060-1707329	+	99%
**E.c.I4-17**	NC_011750	1777316-1779585	+	99%
**E.c.I4-18**	NC_011750	1957302-1959571	+	99%
**E.c.I4-19**	NC_011750	2077903-2080172	+	99%
**E.c.I4-20**	NC_011750	2748227-2750496	+	99%
**E.c.I4-21**	NC_011750	3061174-3063443	-	99%
**E.c.I4-22**	NC_011750	3557284-3559553	-	99%
**E.c.I4-23**	NC_011750	3581215-3583484	+	99%
**E.c.I4-24**	NC_011750	4372956-4375225	+	99%
**E.c.I4-25**	NC_010498	3241200-3243470	+	98%
**E.c.I4-26**	NC_007941	30449-32719	+	99%
	NC_011749	13815-16085	-	99%
**E.c.I4-27**	NC_009790	12712-14982	+	99%
**E.c.I4-28**	NC_009790	23170-25439	-	99%
**E.c.I4-29**	NC_009788	51218-53488	+	99%
**E.c.I4-30**	NC_009786	13831-16100	-	99%
**E.c.I4-tr1**	AC_000091	271415-273178	+	99%
	NC_000913	271415-273178	+	99%
	NC_010473	245519-247282	+	99%
	NC_012759	271414-273177	+	99%
**E.c.I4-tr2**	NC_010498	3240307-3240438	-	84%
**E.c.I4-tr3**	NC_007941	15211-16195	-	99%
	NC_011749	27904-28888	+	99%
**E.c.I4-tr4**	NC_004851	146273-146677	-	84%
	NC_007607	166216-166620	-	84%
	NC_007385	122695-123099	-	85%
	NC_010660	142547-142951	-	84%

Element	Genome	Position	Orientation	Nucleotide Similarity
**E.c.F10a**	NC_004431	4296437-4296613	-	77% (D.a.I1)
**E.c.F10b**	NC_008563	4752376-4752552	+	77% (D.a.I1)
	NC_011742	4667038-4667214	+	77%
**E.c.F11**	NC_009801	3030090-3031244	-	100% (E.c.F11)
**E.c.F12**	NC_004741	3150254-3150371	-	79% (P.s.I1)
	NC_004337	3161591-3161708	-	79%
	NC_007613	2886948-2887065	-	79%
	NC_008258	3135258-3135375	-	79%
	NC_007384	3322476-3322593	-	79%
	NC_009801	3499415-3499532	-	79%
	NC_010658	3213779-3213896	-	79%
	NC_011415	3432487-3432604	-	79%
	NC_011741	3251207-3251324	-	79%
	NC_011748	3518470-3518587	-	79%
**E.c.F13**	NC_011416	45269-45483	+	77% (A.v.I2)
**E.c.F14**	NC_011416	3263-3762	-	92% (P.l.I2)
**E.c.F15**	NC_009788	30548-30682	+	81% (Di.ze.I1)
**S.s.F1**	NC_004851	145845-146276	-	90% (P.l.I2)
	NC_007607	165788-166219	-	89%
	NC_007385	122267-122698	-	90%
	NC_010660	142119-142550	-	90%
**S.s.F2**	NC_004851	157683-158508	-	88% (P.l.I2)
	NC_007385	134260-135695	-	89%
	NC_010660	166256-166978	+	88%
**S.s.F3**	NC_007607	90701-90956	-	87% (P.l.I2)
	NC_007385	162435-162690	-	87%
**S.b.F1**	NC_004851	82167-82315	-	79% (A.v.I1)
	NC_007608	63856-64004	-	79%
	NC_010660	70536-70684	-	79%
**S.b.F2**	NC_004851	90922-91037	-	76% (Ce.ja.I1)
	NC_007608	34843-34958	-	77%
	NC_010660	40984-41099	-	77%
**S.b.F3**	NC_007608	34493-34688	+	77% (Pr.ae.I5)
	NC_010660	41254-41449	-	77%
**S.d.F1**	NC_007607	91024-91568	-	82% (Di.ze.I1)
	NC_007385	162758-163302	-	82%
**S.f.F4**	NC_004851	91343-91479	-	75% (Dh.re.I1)
**S.f.F5**	NC_004851	91611-91745	-	79% (UA.I3)
**S.ma.I1-tr1**	NC_011743	6501-7367	-	99%

Yersinia

Element	Genome	Position	Orientation	Nucleotide Similarity
**Y.p.F1**	NC_003134	76439-77581	-	93% (E.c.I4)
	NC_004838	54417-55559	+	93% (E.c.I4)
	NC_005815	100480-101622	-	93% (E.c.I4)
	NC_008118	82559-83701	+	93% (E.c.I4)
	NC_008120	78113-79255	+	93% (E.c.I4)
	NC_009378	118651-119793	+	93% (E.c.I4)
	NC_010158	96211-97353	+	93% (E.c.I4)

Element	Genome	Position	Orientation	Nucleotide Similarity
**Y.e.F1**	NC_008791	40232-40794	-	91% (E.c.I4)

Klebsiellla

Element	Genome	Position	Orientation	Nucleotide Similarity
**Kl.pn.I5-1**	NC_009649	119263-121172	+	99%
**Kl.pn.I5-2**	NC_009648	1330932-1332841	+	99%

Element	Genome	Position	Orientation	Nucleotide Similarity
**Ps.st.I2-tr1**	NC_009649	40012-40231	+	98%

Element	Genome	Position	Orientation	Nucleotide Similarity
**Kl.pn.F1**	NC_009648	3724567-3725502	-	100% (Kl.pn.F1)
**Kl.pn.F2**	NC_012731	597905-599823	-	100% (Kl.pn.F2)

Dickeya

**Di.da.I1**	NC_012880	814296-816881	-	100%
	NC_012912	788411-790997	-	100%

**Di.ze.I1-1**	NC_012912	3974819-3976683	-	100%
**Di.ze.I1-2**	NC_012912	4627726-4629590	+	100%

Photorabdus

**P.l.I1-1**	NC_005126	1201822-1203729	+	100%
**P.l.I1-tr1**	NC_005126	1273077-1274340/	-	98/96%
		1276223-1276526		
**P.l.I1-tr2**	NC_005126	1300636-1300806	-	94%
**P.l.I1-tr3**	NC_005126	1724220-1724463	-	95%

**P.l.I2-1**	NC_005126	1274341-1276222	+	100%

**P.l.F1**	NC_005126	5072575-5072755	-	87% (Ci.ro.I2)
	NC_012962	4555061-4555319	-	88% (Ci.ro.I2)
**P.l.F2**	NC_005126	5079759-5080083	-	81% (Di.ze.I2)
**P.l.F3**	NC_005126	5080116-5080297	-	90% (Ci.ro.I2)
**P.l.F4**	NC_005126	2837265-2837404	+	75% (Zu.pr.I1)

Serratia proteomaculans

**Se.pr.F1**	NC_009832	4593443-4594420	-	100% (Se.pr.F1)
**Se.pr.F2**	NC_009832	4591663-4591858	-	95% (Se.pr.F1)
**Se.pr.F3**	NC_009832	1068281-1068398	+	76% (Kl.pn.F2)

Sodalis glossinidius

**So.gl.I1-1**	NC_007712	144267-144782/	+	95/97/96%
		144781-145649 /		
		145819-145985		
**So.gl.I1-2**	NC_007712	1926091-1927697	-	99%
**So.gl.I1-3**	NC_007712	2034600-2036252	+	100%
**So.gl.I1-tr1**	NC_007712	1047315-1047755/	+	94/96%
		1047752-1048116		
**So.gl.I1-tr2**	NC_007712	2755590-2756076	-	97%

**So.gl.F1**	NC_007712	4061384-4061762	+	87% (A.v.I2)
**So.gl.F2**	NC_007712	2754745-2755590	+	75% (A.v.I4)
**So.gl.F3**	NC_007713	56167-56445	+	80% (P.l.I1)

Hamiltonella

**c-Ha.de.I1-1**	NC_012751	262660-265005	-	95%
**c-Ha.de.I1-2**	NC_012751	301656-304001	+	100%
**c-Ha.de.I1-3**	NC_012751	356733-359077	-	100%
**c-Ha.de.I1-4**	NC_012751	444534-446879	+	100%
**c-Ha.de.I1-5**	NC_012751	538165-540510	-	100%
**c-Ha.de.I1-6**	NC_012751	567898-570243	-	99%
**c-Ha.de.I1-7**	NC_012751	609654-611999	+	100%
**c-Ha.de.I1-8**	NC_012751	717352-719697	-	100%
**c-Ha.de.I1-9**	NC_012751	720917-723262	-	99%
**c-Ha.de.I1-10**	NC_012751	725420-727762	-	93%
**c-Ha.de.I1-11**	NC_012751	738773-741118	-	99%
**c-Ha.de.I1-12**	NC_012751	853250-855595	+	100%
**c-Ha.de.I1-13**	NC_012751	973312-973896/	-	100/99%
		976243-978002		
**c-Ha.de.I1-14**	NC_012751	973897-976241	+	100%
**c-Ha.de.I1-15**	NC_012751	982403-984748	+	99%

**c-Ha.de.I1-16**	NC_012751	1073294-1075639	+	100%
**c-Ha.de.I1-17**	NC_012751	1132556-1134902	+	99%
**c-Ha.de.I1-18**	NC_012751	1155626-1157971	+	99%
**c-Ha.de.I1-19**	NC_012751	1273155-1275500	+	100%
**c-Ha.de.I1-20**	NC_012751	1299508-1301850	+	93%
**c-Ha.de.I1-21**	NC_012751	1304020-1306365	+	100%
**c-Ha.de.I1-22**	NC_012751	1642570-1644915	-	99%
**c-Ha.de.I1-23**	NC_012751	1771605-1773951	+	99%
**c-Ha.de.I1-24**	NC_012751	1823495-1825255/	+	100/100%
		1827601-1828185		
**c-Ha.de.I1-25**	NC_012751	1825255-1827600	-	100%
**c-Ha.de.I1-26**	NC_012751	1846199-1846784/	-	99/99%
		1849130-1850890		
**c-Ha.de.I1-27**	NC_012751	1846785-1849130	+	100%
**c-Ha.de.I1-28**	NC_012751	2089107-2091452	+	99%
**c-Ha.de.I1-29**	NC_012752	18131-20476	+	100%

**c-Ha.de.I1-tr1**	NC_012751	790930-793141	-	100%
**c-Ha.de.I1-tr2**	NC_012751	1004731-1004859	-	95%
**c-Ha.de.I1-tr3**	NC_012751	1198875-1200743	+	100%
**c-Ha.de.I1-tr4**	NC_012751	1233834-1234418	+	100%
**c-Ha.de.I2-1**	NC_012751	935004-936265/	+	100/100%
		937173-938352		
**c-Ha.de.F1**	NC_012751	1098031-1098177	+	100% (c-Ha.de.F1)
**c-Ha.de.F2**	NC_012751	1828817-1828961	+	88% (c-Ha.de.F1)
**c-Ha.de.F3**	NC_012751	2011450-2011594	+	85% (c-Ha.de.F1)
**c-Ha.de.F4**	NC_012751	1421503-1421628	+	83% (E.c.I8)
